# Evaluation of Spike Protein Epitopes by Assessing the Dynamics of Humoral Immune Responses in Moderate COVID-19

**DOI:** 10.3389/fimmu.2022.770982

**Published:** 2022-03-18

**Authors:** Lingyun Chen, Pengfei Pang, Huan Qi, Keqiang Yan, Yan Ren, Mingliang Ma, Ruyin Cao, Hua Li, Chuansheng Hu, Yang Li, Jun Xia, Danyun Lai, Yuliang Dong, Hewei Jiang, Hainan Zhang, Hong Shan, Shengce Tao, Siqi Liu

**Affiliations:** ^1^ College of Life Science, University of Chinese Academy of Sciences, Beijing, China; ^2^ Department of Proteomics, Beijing Genomics Institution, Shenzhen, China; ^3^ Center for Interventional Medicine, The Fifth Affiliated Hospital of Sun Yat-sen University, Zhuhai, China; ^4^ Shanghai Center for Systems Biomedicine, Shanghai Jiaotong University, Shanghai, China; ^5^ State Key laboratory for Oncogenes and Bio-ID Center, School of Biomedical Engineering, Shanghai Jiao Tong University, Shanghai, China

**Keywords:** SARS-CoV-2, COVID-19, S protein, epitope, dynamics, ELISA, microarray, AbMap

## Abstract

The coronavirus disease 2019 (COVID-19) pandemic is caused by a novel coronavirus called severe acute respiratory syndrome coronavirus 2 (SARS-CoV-2). The spike protein (S) of SARS-CoV-2 is a major target for diagnosis and vaccine development because of its essential role in viral infection and host immunity. Currently, time-dependent responses of humoral immune system against various S protein epitopes are poorly understood. In this study, enzyme-linked immunosorbent assay (ELISA), peptide microarray, and antibody binding epitope mapping (AbMap) techniques were used to systematically analyze the dynamic changes of humoral immune responses against the S protein in a small cohort of moderate COVID-19 patients who were hospitalized for approximately two months after symptom onset. Recombinant truncated S proteins, target S peptides, and random peptides were used as antigens in the analyses. The assays demonstrated the dynamic IgM- and IgG recognition and reactivity against various S protein epitopes with patient-dependent patterns. Comprehensive analysis of epitope distribution along the *spike* gene sequence and spatial structure of the homotrimer S protein demonstrated that most IgM- and IgG-reactive peptides were clustered into similar genomic regions and were located at accessible domains. Seven S peptides were generally recognized by IgG antibodies derived from serum samples of all COVID-19 patients. The dynamic immune recognition signals from these seven S peptides were comparable to those of the entire S protein or truncated S1 protein. This suggested that the humoral immune system recognized few conserved S protein epitopes in most COVID-19 patients during the entire duration of humoral immune response after symptom onset. Furthermore, in this cohort, individual patients demonstrated stable immune recognition to certain S protein epitopes throughout their hospitalization period. Therefore, the dynamic characteristics of humoral immune responses to S protein have provided valuable information for accurate diagnosis and immunotherapy of COVID-19 patients.

## Introduction

The coronavirus disease 2019 (COVID-19) pandemic is caused by a novel and highly contagious and pathogenic coronavirus (CoV) called severe acute respiratory syndrome coronavirus 2 (SARS-CoV-2) (1). To date, seven human CoVs, namely hCoV-NL63, hCoV-229E, hCoV-OC43, hCoV-HKU1, severe acute respiratory syndrome CoV (SARS-CoV), Middle East respiratory syndrome CoV (MERS-CoV), and SARS-CoV-2 have been identified and characterized (2, 3). SARS-CoV, MERS-CoV, and SARS-CoV-2 infections can cause life-threatening diseases with strong pandemic potential (4). Multiple factors, including host immunity against viral infection influence COVID-19 diagnosis and therapy (5–7). Therefore, the characterization of humoral immune responses against SARS-CoV-2 would greatly advance the development of novel diagnostic approaches and effective vaccines.

The innate or adaptive immune responses of the host that are elicited upon encountering SARS-CoV-2, generate detectable SARS-CoV-2-specific antibodies between 10 and 14 days after symptom onset (8–11). The identification of viral antigenic epitopes that induce humoral immune responses is essential for understanding host immunity against SARS-CoV-2. As previously observed with other coronaviruses, SARS-CoV-2 genome-encoded *spike* (*S*) and *nucleocapsid* (*N*) gene expression products are highly immunogenic and major targets of antibodies (12, 13). Hence, both these antigens are relevant for the diagnosis of COVID-19 and form the basis for most immunoassays available in the clinic (14, 15). In contrast to the nucleocapsid (N) protein, the spike (S) protein is not only the main causal factor of immunogenicity, but also plays a central role in viral entry into host cells by binding to angiotensin-converting enzyme 2 (ACE2) (16). Zhou et al. reported that convalescent serum against S protein was both a marker for viral exposure and an indicator of recovery from viral infection (17). Dispinseri et al. claimed a strong correlation between IgG antibodies against the S protein of COVID-19 and viral neutralization (18). Therefore, the S protein is the primary focus of studies related to SARS-CoV-2 vaccines and antibody-based therapeutics.

The immunogenic characteristics of the S protein from SARS-CoV-2 are well known. Poh et al. reported that two linear S epitopes elicited the neutralizing antibodies (19). Shrock et al. showed the IgA and IgG recognition of immunodominant regions in S protein (20). Recently, some studies reported temporal changes in the humoral immune response after symptom onset (21–25). Ravichandran et al. performed a comprehensive longitudinal analysis of the antibody repertoire to S protein in COVID-19 patients during their hospital stay between the second and tenth weeks and demonstrated a correlation between increased antibody affinity maturation to prefusion COVID-19 S protein and disease severity (23).

Effective immunity against viral infection relies on the ability of B cells to generate a diverse repertoire of antibodies to neutralize the virus (26). Activated B cells form germinal centers in the secondary lymphoid tissues (spleen and peripheral lymph nodes) after encountering the virus and undergo iterative cycles of clonal expansion and somatic hypermutations in the variable regions of their immunoglobulin heavy and light chain genes, followed by affinity-based selection of antibodies with high antigen specificity (27). Moreover, recent high-throughput sequencing technologies have shown novel perspectives regarding the generation of B cell receptor (BCR) repertoires in a time- and individual-dependent manner, which orchestrate dynamic humoral immune responses against influenza virus, Zika virus (28), Ebola virus (29), and HIV (30). As for SARS-CoV-2, BCR repertoire sequencing revealed the usage frequency of different *V* and *J* gene segments and B-cell clonal expansion in infected individuals during the period after symptom onset (31–34). Nielsen et al. reported extensive class switching to IgG and IgA subclasses with limited hypermutations during the initial weeks of COVID-19 infection (31).

Several research groups are currently attempting to understand the mechanisms underlying the role of immunoglobulin gene editing or immunoglobulin recognition in the S protein of SARS-CoV-2. Most reports in this research area have relied on data generated from a single technology; therefore, the relevant conclusions have lacked supporting evidence through different technologies. In addition, early studies did not focus on designing and analyzing the general patterns of longitudinal recognition of immunoglobulins to S epitopes, while the scattered reports from different investigators were difficult to integrate for a fundamental understanding of the time-dependent rule of humoral immune responses against SARS-CoV-2. These prompted us to initiate a project, which carried out a systematical survey to the longitudinal changes of humoral immune responses specifically against S epitopes. A total of 123 serum samples from 19 patients with COVID-19 were collected over a period of approximately two months after symptom onset. The time-dependent reactivity of immunoglobulins in patients was assessed using three types of antigens *in vitro*: recombinant truncated S proteins, synthesized S peptides, and random peptides. The experimental design and data analysis are illustrated in **Supplementary Figure 1**.

## Materials and Methods

### Collection of Serum Samples From Moderate COVID-19 Patients

Nineteen COVID-19 patients were recruited, who were admitted to the Fifth Affiliated Hospital of Sun Yat-sen University and were clinically treated according to the Diagnosis and Treatment Protocol for Novel Coronavirus Pneumonia (Trial Version 4 released by the National Health Commission & State Administration of Traditional Chinese Medicine on January 27, 2020). All COVID-19 patients were positive for SARS-CoV-2 according to the RT-PCR results from oropharyngeal swabs and showed moderate COVID-19 disease symptoms. All patients had been hospitalized after symptom onset and blood samples were collected during hospitalization. Considering the common cases of COVID-19 patients and comparable treatments in the hospital, 123 blood specimens were collected in a time-interval mode from these 19 patients during the hospitalization period of approximately two months, starting on February 1st and ending on March 29th (**Supplementary Tables 1**, **2**). The average age of the patients was 51 years (range: 29–71; 9 women and 10 men). As the control group, the non-COVID-19 sera were donated from 27 healthy donors whose blood samples were collected from the same hospital. Blood specimen collection was approved by the Research Ethics Committee of the Fifth Affiliated Hospital of Sun Yat-sen University, Zhuhai, China (Approval No. K62-1), and signed written informed consent was obtained from all the participants of the study.

### Estimation of Humoral Immune Responses Against SARS-CoV-2 by Enzyme Linked Immunosorbent Assay (ELISA)

Serum antibodies were analyzed in COVID-19 patients and healthy subjects using the commercial ELISA kits. Serum IgG activity against purified antigens of inactivated viral lysates was measured using the SARS-CoV-2 Virus IgG Antibody Detection Kit (Beijing BGI-GBI Biotech Co., Ltd., Beijing, China). Serum IgM activity against recombinant S1 and N proteins with IgM µ-chain capture was measured using the SARS-CoV-2 Virus IgM Antibody Detection Kit (Beijing BGI-GBI Biotech Co., Ltd., Beijing, China).

### Microarray Analysis of Humoral Immune Responses Against SARS-CoV-2

#### Microarray Construction

The *S* gene sequence (MN908947.3) of SARS-CoV-2 was downloaded from the GenBank database. The *S* gene fragments corresponding to *S1*, *RBD*, and *S2* were synthesized (Sangon Biotech, Shanghai, China) and cloned into the pGEX-4T-1 vector. The expression vector was transformed into *Escherichia coli* BL21 for the expression of the recombinant S1, S2, and RBD, and the expressed proteins were purified using GST-Sepharose beads (Senhui Microsphere Technology, Suzhou, China (35). The 12-mer linear peptides covering the entire S protein sequence (1–1,273, YP_009724390.1) were designed based on the interval overlap of six residues, and in total of 211 peptides with N-terminal amidated were chemically synthesized (GL Biochem, Ltd., Shanghai, China). These S peptides were conjugated with BSA using Sulfo-SMCC (Thermo Fisher Scientific, MA, USA) according to the instructions of the manufacturer. The S recombinants and synthesized peptides were printed in triplicate onto PATH substrate slides (Grace Bio-Labs, Oregon, USA) using the Super Marathon printer (Arrayjet, Roslin, UK) to generate identical arrays in a 1 × 7 subarray format (36). The microarrays were stored at −80°C until further use. To normalize the fluorescence signals in the microarray, GST, biotin-control, and eGFP were used as negative controls, while human IgG, human IgM, and ACE2-Fc as positive controls.

#### Microarray-Based Immunoassay

A 14-chamber rubber gasket was mounted on each slide to create individual chambers with 14 identical homemade subarrays. The previously frozen arrays were warmed to room temperature and incubated in the block buffer (3% BSA in 1 × PBS buffer with 0.1% Tween 20) for 3 h. The serum samples were diluted with 1× PBS containing 0.1% Tween 20 (1:200) and incubated with each subarray for 2 h at room temperature. After washing with 1× PBST, the subarrays were incubated with secondary antibodies, namely Cy3-conjugated goat anti-human IgG and Alexa Fluor 647-conjugated donkey anti-human IgM (Jackson ImmunoResearch, PA, USA) at room temperature for 1 h. Subsequently, the subarrays were washed with 1× PBST again, dried at room temperature by centrifugation, and scanned using LuxScan 10 K-A (CapitalBio Corporation, Beijing, China) with the following parameters: 95% laser power/PMT 550 for IgM and 95% laser power/PMT 480 for IgG.

#### Microarray Data Processing

The fluorescence intensities (FI) from the microarray were extracted using the GenePix Pro 6.0 software (Molecular Devices, CA, USA). For each spot, the FI was obtained by subtracting the FI of the background from that of the foreground. The FI quantification of humoral immune responses to the individual recombinant S proteins or peptides was performed by calculating the average of FI from triplicate spots. The positive peptides were recognized from the COVID-19 sera by using a cut-off value of mean FI + 3 × standard deviation (SD) of healthy subjects. The intensity of the immune reactivity for each peptide was normalized in different patients using the Z-score, which was calculated as follows: *Z score* = *FI_Ppn_* – *meanFI_Pp_*_1…_ *_Ppn_*)/*SD_Pp_*_1_ *_…Ppn_*, where *Ppn* is defined as the peptide or protein reactivity at a sampling point from a COVID-19 patient and *Pp1*…*Ppn* represents cumulative measurements of all sampling points from the same COVID-19 patient (37).

### AbMap Analysis of Humoral Immune Responses Against SARS-CoV-2

#### Purification of Antibodies Against S1 Protein in the Patient Sera

Recombinant S1 protein (Sino Biological, Beijing, China) was biotinylated according to the protocol of the manufacturer (Thermo Fisher Scientific, Rockford, USA). The biotinylated S1 protein was then incubated with Dynabesads™ Myone™ Streptavidin T1 (Thermo Fisher Scientific, Carlsbad, USA) at room temperature for 1 h to immobilize the protein on the surface of the magnetic beads (S1-magnetic beads). Then, the serum samples from COVID-19 patients were incubated with the S1-magnetic beads at 4°C for 4 h. Then, the S1-magnetic beads were washed with PBST to eliminate non-specific binding. The bound antibodies were eluted with 50 mM glycine (pH 2.8) followed by neutralization with 1M Tris buffer (pH 8.0).

#### AbMap Assay

The antibody binding epitope mapping (AbMap) assay developed in our laboratory was previously performed (38). Briefly, 96-well PCR plates were blocked with PBST containing 3% BSA at 4°C for 16 h. Each well was then loaded with Ph.D.-12 phage display libraries (New England Biolabs, MA, USA) followed by adding the S1 antibody purified above. The mixtures were incubated at 4°C for 16 h. Dynabeads™ Protein G (Thermo Fisher Scientific, Carlsbad, CA, USA) was added into each well to capture the antibody and phage complex at 4°C for 4 h. The magnetic beads in each well were collected and washed. The beads suspended in water were boiled at 98°C for 10 min, and the resulting supernatant was collected for further PCR analysis.

To introduce the adapter sequence and unique barcode or index for each sample, two rounds of PCR were carried out on the phage lysate using Q5 hot-start polymerase. The first round of PCR was performed by using XX-S5XX-23R and XX-N7XX-18 primers (5′-TCGTCGGCAGCGTCAGATGTGTATAAGAGACAGXXXXXXXXGTGGTACCTTTCTATTCTCACTCT-3′, and 5′-GTCTCGTGGGCTCGGAGATGTGTATAAGAGA CAGXXXXXXXXTTCAACAGTTTCGGCCGAACCT-3′, respectively; where, “XXXXXXXX” denotes an eight-nucleotide barcode sequence; the sequence with the underline represents the specific primer for amplifying the corresponding nucleotides of the displayed peptides from the phage genome; the remaining sequence represents the Illumina index). After electrophoresis, all PCR products were mixed and purified as templates for the second round of PCR. In the second round of PCR, unique indices of Illumina next generation sequencing (NGS) were introduced for each mixture. The products obtained from the second round of PCR were sequenced using Illumina HiSeq 2000 (Illumina Inc. CA, USA).

#### AbMap Data Processing

The NGS results were split and assigned to each sample based on the index and barcode combinations. For each sample, the NGS data were trimmed further and only sequences of 36 base pairs corresponding to the 12-mer displayed peptides remained. All the remaining sequences were translated into peptides and the translation frequency of each peptide was counted. The enrichment and reverse enrichment factors for each peptide from the samples were calculated and set as cutoff values. The peptides with the enrichment factors above the cutoff were retained for subsequent motif analysis. The remaining peptides were subjected to MEME (Motif-based Sequence Analysis Tools, https://techtransfer.universityofcalifornia.edu/NCD/20911.html) to identify motifs that represent clusters of 12-mer peptides. During this analysis, eight motifs were generated for each sample and a motif with an E value less than 0.01 was considered significant and further matched to the S protein sequence using the MAST (Motif Alignment & Search Tools, https://mccb.umassmed.edu/meme/doc/mast.html).

### Dynamic Immune Response Data Analysis

Since the time points of specimen collection varied between different patients in this study, the dynamic data were normalized according to the weeks after symptom onset during hospitalization. For individual patients, dynamic analysis was performed on the immune responses against different S proteins or epitopes that were consistently observed during the hospitalization weeks and normalized by Z-scores. The dynamic analysis included (1) estimating positive frequencies of immune recognition in all patients for individual peptides from the microarray or AbMap, (2) hierarchically clustering the quantified immune responses from the microarray, and (3) assessing the dynamic behaviors of the S proteins or peptides commonly observed in the patient sera by (1) statistical curve fitting of the normalized intensities of immune responses at different time points and (2) generating heatmaps with intensities of immune responses. All statistical analyses were performed using R statistical software.

### Spatial Analysis of the S Epitopes

The secondary structures of the potential S epitopes were analyzed by DPSS (Dictionary of Protein Secondary Structure, https://2struc.cryst.bbk.ac.uk/about/). The 3D structure of the S protein from Zhang’s laboratory (https://zhanglab.ccmb.med.umich.edu) was taken to analyze the spatial location of the potential epitopes. All the spatial images were processed using the PyMOL software (The PyMOL Molecular Graphics System, Version 2.0, Schrödinger, NY, USA).

## Results

### Dynamics of Humoral Immune Responses Against S Proteins in the COVID-19 Patients

**Figure 1:**
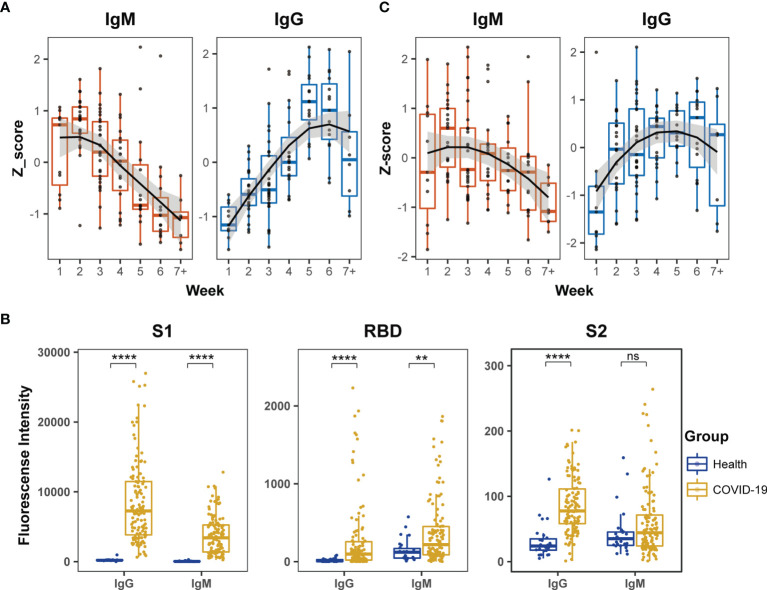
Dynamic behaviors of humoral immune responses against S protein in COVID-19 patients. **(A)** The dynamic changes of IgM against N and recombinant S1 protein and IgG against the extract proteins from virus lysate based on ELISA. The signals of antibody responses in the patient serum samples were normalized by Z-scores and the trends of signal changes were mimicked by curve fitting. **(B)** Comparisons of IgM or IgG immune signals against the truncated S proteins, S1, RBD and S2, between healthy subjects (n = 27) and all COVID-19 (n = 123) serum samples based on the microarray data. Unpaired Student’s t-test was used in the statistical analysis. Note: **p < 0.01, ****p < 0.0001, and ns, non-significance. **(C)** The dynamic changes of IgM and IgG against recombinant S1 protein based on microarray.

First, the humoral immune responses to SARS-CoV-2 in COVID-19 patients were estimated by ELISA, and the levels of specific IgM and IgG antibodies were measured using N and recombinant S1 proteins and viral extracts as antigens. The Z-scores of IgM against the N and recombinant S1 protein were significantly high during the first two weeks after symptom onset and then gradually diminished during the remaining period of hospitalization (**Figure 1A**). In contrast, the Z-scores of IgG against the viral extracts remained in a continuously increased mode during the first four weeks and reached a plateau between the fifth and sixth weeks after symptom onset (**Figure 1A**).

Next, the humoral immune responses against SARS-CoV-2 were evaluated by microarray using three recombinant truncated S proteins: S1, S2 and RBD. The serum samples of COVID-19 patients exhibited significantly higher IgG antibody reactivity against all three recombinant S proteins than healthy subjects (**Figure 1B**). Moreover, the strength of serum antibody reactivity varied significantly between individual recombinant S proteins. The recombinant S1 protein showed 10-fold higher serum antibody reactivity than the recombinant S2 and RBD proteins (**Figure 1B**). In the COVID-19 patients, serum IgM reactivity was generally lower than the corresponding serum IgG reactivity against the recombinant S proteins (**Figure 1B**). Furthermore, although IgM reactivity signals against RBD in COVID-19 patients were significantly higher than those in healthy subjects, the signals were relatively low among the COVID-19 patients and did not provide reliable dynamic data. The IgM antibodies in the COVID-19 patients displayed poor reactivity against S2 and the signal was similar to that displayed by healthy subjects (**Figure 1B**). These findings demonstrated much stronger affinity of the patient serum samples against S1 than RBD and S2 recombinant proteins in the microarray assay. In further dynamic analysis of humoral immune responses to S-truncated proteins, S1 was selected as the main immune target but not S2 and RBD. The time-dependent serum IgM and IgG reactivity against S1 in all patients were plotted in **Figure 1C**, in which the trends of immune reactivity were similar to the ELISA data illustrated in **Figure 1A**; IgM activity emerged at an early time point and subsided, whereas IgG activity emerged at a later time point and was sustained for longer. As the time-dependent pattern of IgG reactivity to the intact proteins in the extract of virus-infected cells (**Figure 1A**) was similar to the pattern derived from **Figure 1C**, these data suggested that the humoral immune responses of COVID-19 patients mainly targeted the S1 protein compared to other viral antigens.

### Evaluation Towards the Epitope Features of S Peptides Recognized by the COVID-19 Sera on Microarray

**Figure 2:**
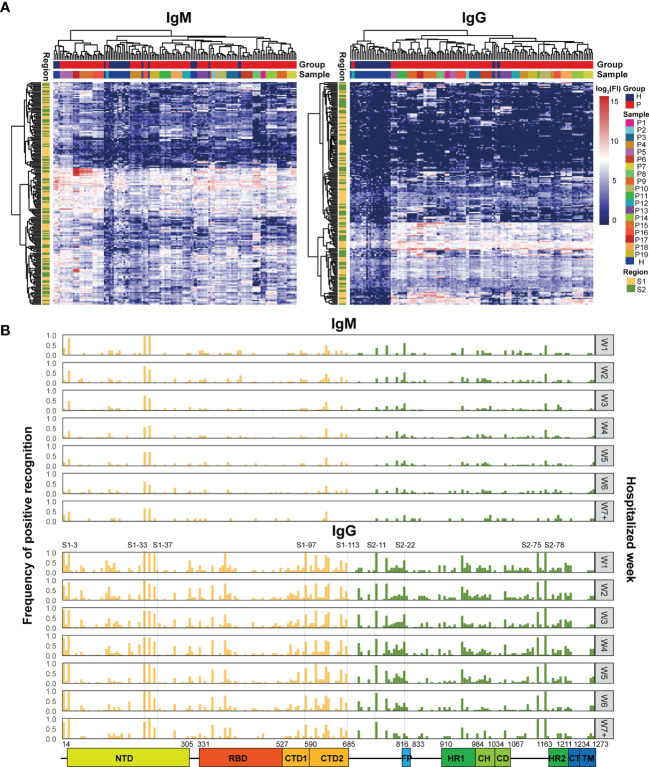
Microarray analysis of the immune recognition features in the S peptide epitopes. **(A)** Hierarchical clustering of signals based on immune reactivity from IgM or IgG of healthy subjects and COVID-19 patients against all the S synthesized peptides measured by microarray. The bars upper: distribution of the healthy (blue) and COVID-19 (red) samples (top) and of all the individual samples (bottom) based on the clustered results. The bars right: the intensity indicator of immune reactivity (left) and the indicator of individual samples (right). The bar left indicates the distribution of S1 (yellow) and S2 (green) based on the cluster results. **(B)** Frequency distribution of the S peptides positively recognized by the patient IgM or IgG during hospitalization. The x axis represents the entire *S* gene sequence; the color bars at the bottom denote functional domains of S protein, namely, N-terminal domain (NTD, 14–305), receptor binding domain (RBD, 331–527), C-terminal domain 1 (CTD1, 528–590), C-terminal domain 2 (CTD2, 591–684), fusion peptide (FP, 816–83), heptad repeat 1 (HR1, 910–984), central helix (CH, 985–1034), connector domain (CD, 1,035–1,067), HR2 (heptad repeat 2, 1,163–1,211), transmembrane domain (TM, 1,212–1,234), and cytoplasmic tail (CT, 1,235–1,273).The y axis (left) represents the frequency of the S peptides that are positively recognized by antibodies, while the gray signs on right mean the hospitalization time (weekly counted). The yellow and green bars indicate S peptides located in the S1 and S2 subunits, respectively.

To further study the dynamic features of humoral immune responses to S epitopes during the period of hospitalization, a peptide microarray comprising 211 peptides derived from the S protein was implemented to assess the immune reactivity between the peptides and the patient sera. Hierarchical clustering analysis illustrated that serum IgM reactivity against the S peptides were similar in COVID-19 patients and healthy individuals, few serum samples from COVID-19 patients gave positive signals (**Figure 2A**). However, serum IgG from COVID-19 patients showed higher reactivity against some S peptides than healthy individuals (**Figure 2A**). Furthermore, the signals from both IgM and IgG antibodies for individual patient samples at multiple time points were clustered together (**Figure 2A**). This suggested generation of highly specific and unique antibodies in individual patients against the S peptides.

After applying strict criteria (mean + 3 SD of the signal in healthy subjects) to remove S peptides with weak immune signals, 124 IgM-reactive S peptides and 165 IgG-reactive S peptides were identified in COVID-19 patients. The number of S peptides recognized by the serum samples was patient-dependent, with 1–45 IgM-reactive peptides and 38–91 IgG-reactive peptides per patient (**Supplementary Figure 2**). The peptides uniquely recognized by individual patients occupied relatively higher ratios: 42.7% (53/124) for IgM and 16.4% (27/165) for IgG. Specifically, none of the S peptides (0/124) were commonly recognized by IgM antibodies in the sera of all 19 patients, whereas 10 (6%; 10/165) S peptides were commonly recognized by IgG antibodies in the serum samples of all 19 COVID-19 patients. These results implied that the S epitopes commonly recognized by patient serum samples were quite limited, even for IgG antibodies. On the other hand, IgM reactivity against the S peptides was very weak in the patient serum samples, and therefore accounted for greater diversity in the recognition of the S peptides between COVID-19 patient serum samples.

The question was how the S peptides recognized by COVID-19 IgM or IgG antibodies were localized along the viral genomic sequences. The frequencies of S peptide recognition by IgM or IgG antibodies in all patients during the consecutive periods of hospitalization were plotted against the *S* gene regions in the SARS-CoV-2 genome, as shown in **Figure 2B** (Top: IgM; Bottom: IgG). The S peptides reacting with higher frequencies against patient IgG were mainly present in four regions, namely, residues 193–228 in NTD (S1-33 to S1-37), residues 577–684 in CTD (S1-97 to S1-113), 746–829 in S2C1 adjacent FP (S2-11 to S2-22) and 1,130–1,219 in HR2 and TM (S2-75 and S2-88). Although IgM antibodies recognized fewer S peptides with high affinity, those that were highly reactive and frequent were also distributed in the same four regions (**Figure 2B**). The epitopes of the S protein corresponding to those reactions with the IgM and IgG antibodies from different COVID-19 patients were clustered to similar genomic regions, even though the recognition specificity and reactivity varied significantly among the COVID-19 patients. The recognition frequencies of many peptides, including those from the four regions mentioned above, progressively decreased during the later stages of hospitalization. This suggested that the humoral immune responses to epitopes in a population were further diverse after symptom onset. Overall, the S peptide microarray analysis results demonstrated that the reactivity of the S peptides was significantly weaker for IgM antibodies than for IgG antibodies in all COVID-19 patients. Moreover, some IgM- and IgG-specific S peptides showed similar genomic distributions in the *S* gene. In addition, if the IgG-specific S peptides with 50% frequency in the COVID-19 patients (M50) were introduced (**Supplementary Table 3**), the peptides of M50 were distributed along S1, RBD and S2 as 4.5, 2, and 2.5 M50 peptides per fragment of hundred amino acids, respectively. This evidence supported the conclusion drawn from the microarray with recombinant S antigens, in which the S1 region occupied more antigenicity sites than RBD and S2.

### Dynamics of Humoral Immune Responses Against S Peptides in the COVID-19 Patients

**Figure 3:**
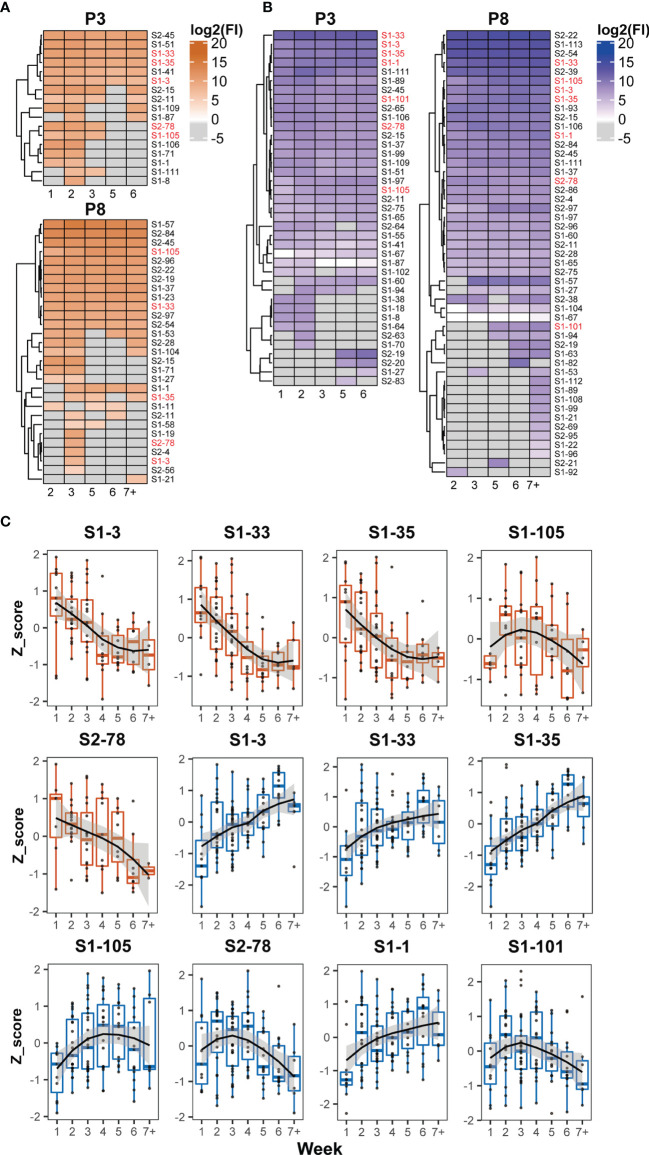
Dynamic patterns of humoral immune responses against reactive S peptides in the COVID-19 patients. Heatmaps of IgM **(A)** and IgG **(B)** against S-reactive peptides during hospitalization in the sera of two COVID-19 patients, P3 and P8. The gray cells on the heatmaps indicate signals with values lower than the cutoffs. The x axis of heatmap indicates the time intervals (week) during the hospitalization after symptom onset. **(C)** The dynamic changes of IgM (boxplot colored in orange) and IgG (boxplot colored in blue) against five IgM-reactive S peptides recognized in at least 50% patients and seven IgG-reactive S peptides recognized in all patients. The signals were normalized by the Z-score and the signal patterns were mimicked by curve fitting.

The recognition status of humoral immune responses to S peptides was individually scrutinized at multiple time points during hospitalization. Based on the threshold setting (mean + 3 SD of the signal in healthy subjects) for the positive detection of the S peptides on the microarray, the S peptides recognized by patient-specific IgM and IgG antibodies could be classified into continuous and discontinuous groups between the first and seventh weeks. The S peptides in the continuous group were defined as detectable recognition signals at alltime points, whereas those in the discontinuous group were not. Microarray analysis showed that 0–24 and 1–37 S peptides were recognized by IgM, and 5–45 and 14–71 S peptides were recognized by IgG in the continuous and discontinuous groups per patient, respectively (**Supplementary Figure 3**). In two representative COVID-19 patients (P3 and P8), 6 and 12 S peptides were continuously recognized by IgM and 25 and 29 S peptides were continuously recognized by IgG (**Figures 3A, B**). Importantly, in the continuous groups, almost all the S peptides recognized by IgM were enclosed within those that reacted with IgG, whereas in the discontinuous group, the majority of the S peptides recognized by IgM did not show reactivity with IgG and vice-versa. These results revealed that recognition of humoral immune responses to certain S peptides was relatively stable in a COVID-19 patient during the first two months after symptom onset. The observation prompted a deduction that once the B cells are matured in response to SARS-CoV-2 infection in an individual, the recognition affinity of the IgM and IgG antibodies to some epitopes is fixed for a long duration after symptom onset. Moreover, the stability of immune recognition is typically individual-dependent.

To analyze whether the humoral immune responses to COVID-19 infection possessed the common recognition to S peptides in this cohort in a longitudinal manner, the S peptides generally recognized by the patient sera were selected based on a cutoff of signal intensity. Five IgM-specific peptides with 50% positive frequency and seven IgG-specific peptides with 100% were identified. Meanwhile, all IgM-specific S peptides with 50% positive frequency completely overlapped with the 100% IgG-specific S peptides detected. The longitudinal changes of the Z-scores for these selected peptides in the corresponding patients were profiled during the entire period of serum collection (**Figure 3C**). The dynamic behaviors of the five IgM-specific peptides were divided into two types: the Z-scores of four peptides (S1-3, S1-33, S1-35 and S2-78) retained the attenuation trends from the first to seventh week, whereas only one peptide (S1-105) exhibited a bell curve with a peak in the third week. The dynamic responses of the patient IgM antibodies against these selected peptides were basically consistent with the time-dependent changes in S1 protein recognition by IgM, as analyzed by ELISA and microarray (**Figures 1A, C**). As for the immune responses against the seven IgG-specific S peptides selected, all patient sera appeared the lowest intensity of immune reaction during the first week after symptom onset. Then, four of the seven IgG-specific peptides (S1-1, S1-3, S1-33 and S1-35) increased continuously until the sixth week, whereas, the remaining three IgG-specific S peptides (S1-101, S1-105 and S2-78) displayed bell-shaped curves with peaks around the second to fourth week. The dynamic behaviors of IgG-specific S peptides were similar to those of ELISA and microarray data, which showed increased IgG reactivity between the second and sixth week (**Figures 1A, C**). Despite variations in the recognition of S peptides by IgM or IgG antibodies, seven IgG-specific S peptides were likely regarded as the typical S epitopes that are commonly recognized by humoral immune response, and their patterns of dynamics coincided with that of the recombinant S1. As stated earlier, the humoral immune responses to the S2 protein at relatively lower extent, thus the seven epitopes mainly from S1 would represent intact S proteins for the study of COVID-19 related immunology.

### Appraisal of the Epitope and Dynamic Features of the Random Peptides Recognized by the COVID-19 Patients Using Abmap

An alternative approach, AbMap, was adopted to further evaluate the dynamic behavior of S epitopes recognized the COVID-19 sera. To acquire antibodies against the S protein from patient sera, the antibodies were individually purified from the patient sera through magnetic beads conjugated with recombinant S1 protein. The purified antibodies were then hybridized with random peptides generated from the phage display peptide pool. DNA sequencing data were used to annotate the coding nucleotides of a peptide, and several annotated peptides with similar structures in their amino acid sequences were termed motifs. Stronger immune interaction between the motif and the corresponding antibody was observed when the peptides were derived from distinct sequences representing a motif. Then, motifs from multiple peptides with similar structures were aligned to the sequences of the S protein and the matched motifs were designated as the S epitope.

**Figure 4:**
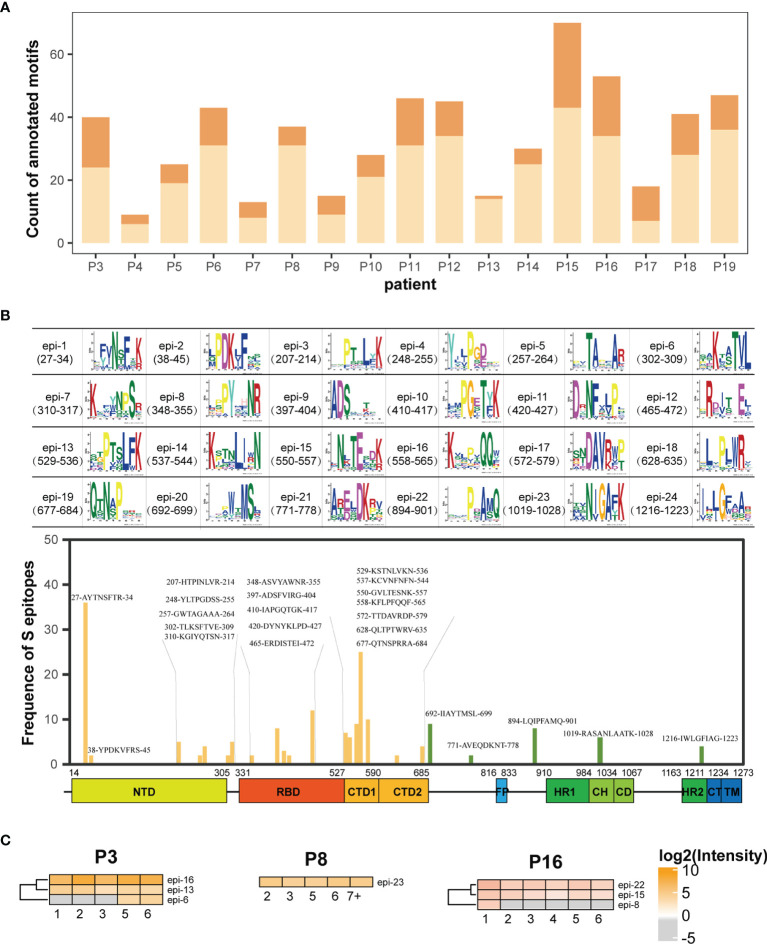
AbMap analysis to identify epitopes with a random peptide library for S1-specific antibodies enriched from the COVID-19 sera. **(A)** Distribution of annotated motifs identified by AbMap in all the COVID-19 patients; motifs matched to the S protein are shown in dark orange and unmatched motifs are shown in light orange. **(B)** Frequency distribution of epitopes recognized by the purified IgG antibodies against the S1 protein in all COVID-19 sera. The upper panel represents 24 structures of matched motifs. The lower panel illustrated frequency distribution of 24 epitopes along the S protein sequence. The x axis represents the entire *S* gene sequence and the color bars at the bottom denote the different domains as indicated in **Figure 2B**. The y axis represents frequency of epitopes detected in the COVID-19 patients. The yellow and green bars indicate epitopes located in the S1 and S2 subunits, respectively. **(C)** Heatmaps of the S epitopes recognized by the purified IgG in the three COVID-19 patients, P3, P8 and P16. The gray cells indicate unmatched S epitopes identified from the corresponding samples. The x axis of heatmap indicates the time intervals (week) during the hospitalization after symptom onset.

Based on motif analysis, 575 motifs were identified from the sera of 19 patients. Among these, 174 motifs matched with the amino acid sequence of the S protein. The distribution of matched and unmatched motifs in individual patients was shown in **Figure 4A**. The matched motifs from 1 (P13) to 34 (P15) were fitted to the 24 S epitopes and ranged from 2 motifs/epitope to 36 motifs/epitope (**Figure 4B**). In addition, AbMap analysis showed that the antibodies from each patient recognized 1–5 S epitopes (**Supplementary Figure 4**). For instance, epi-5 was recognized in only one patient (P11), whereas four epitopes (epi-1, epi-7, epi-14, and epi-18) were commonly recognized in several patients. The matched motifs were unevenly distributed along the *S* gene regions in the SARS-CoV-2 genome for all samples and mainly covered residues 207–317 (NTD), 348–472 (RBD), and 529–579 (CTD) of the S1 protein (**Figure 4B**). The results reached the expectations of the experimental design because the patient IgG would have an affinity binding to the recombinant S1. Moreover, if the number of annotated peptides in an S epitope contributed to a high intensity of immune reactivity, the intensities of all the S epitopes in a patient were clustered during the entire duration of serum collection, thereby allowing the assessment of dynamic humoral immune responses against S epitopes (**Supplementary Figure 4**). As shown in **Figure 4C**, one, three, and four epitopes in P8, P16, and P3 were well-recognized by the patient antibodies, respectively. The dynamic intensities of the immune reactivity against these S epitopes were irregular during hospitalization. Some S epitopes showed continuous positivity, whereas others showed positivity for shorter durations. However, in all 19 patients, at least one S epitope per patient was continuously recognized by the corresponding serum antibodies. The data in **Figure 4C** confirmed the conclusion elicited from **Figures 3A, B** that some recognition specificities of antibodies against the S protein in individual COVID-19 patients were relatively stable after symptom onset in this study.

Assessment of immune recognition against the S epitopes in the COVID-19 patients was implemented using two approaches in parallel: microarray and AbMap. The analysis focused on two aspects: epitope distribution along *S* gene and the dynamic immune reactivity. The number of S epitopes identified through the microarray analysis was higher than those identified through the AbMap analysis; moreover, S epitopes identified by the AbMap analysis overlapped with the microarray data (**Figures 3A, B**, **4C**). Deeply looking at the distribution of the epitopes on the *S* gene, however, there were two S1 epitope regions detected by microarray that overlapped with the same regions on S1 identified by AbMap, whereas the two epitope regions on S2 upon microarray analysis were almost undetected by AbMap (**Figures 2B**, **4B**). This result was expected because we purified the IgGs for AbMap based on their affinity binding to the recombinant S1 protein, which showed poor overlap with S2. Carefully checking the dynamic responses of the S epitopes, the immune recognition of either the commonly shared or individual unique epitopes appeared to be inconsistent. However, the dynamic behavior of some S epitopes on AbMap was in agreement with the microarray observations, recognition specificity, and reaction intensity in an individual consistently lasting for a relatively long period after symptom onset. These results demonstrated that the microarray and AbMap data were reasonably comparable and complementary.

### Spatial Characteristics of the S Epitopes Recognized by COVID-19 Serum Antibodies

**Figure 5:**
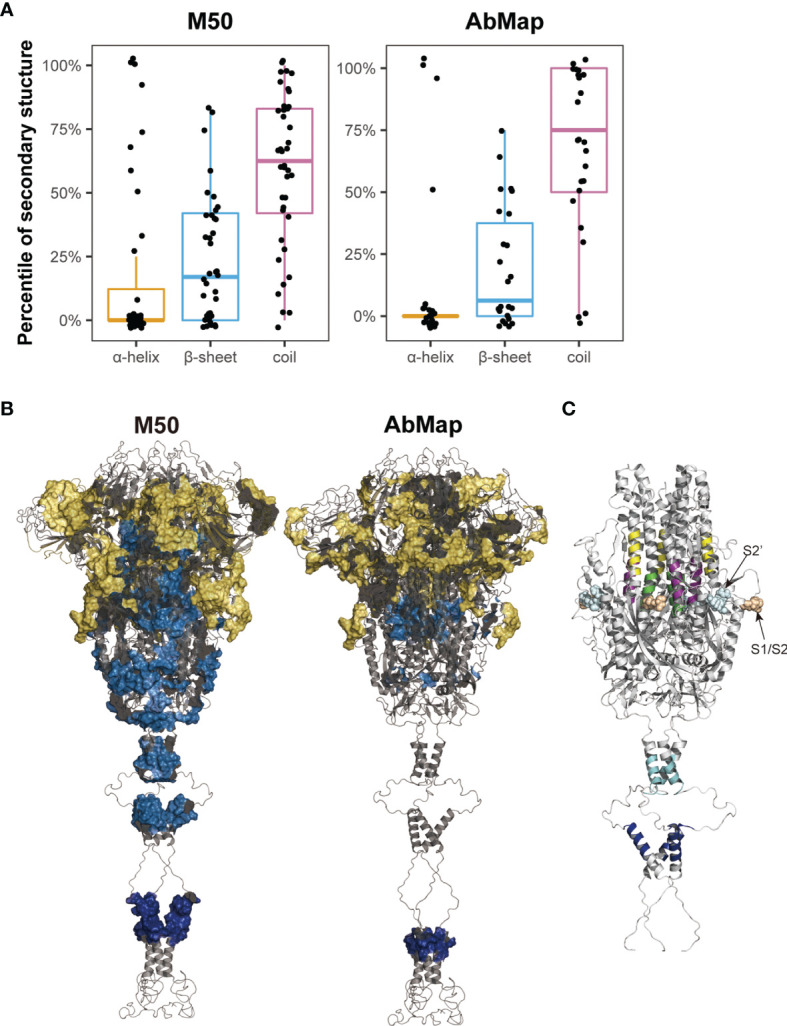
Spatial characteristic of the epitopes on S protein. **(A)** Distribution of secondary structures (α-helix, β-sheet, and random coil) analyzed by DSSP in M50 S peptides based on the microarray and 24 epitopes identified by AbMap. The y axis represents the percentiles of secondary structures in each of the S epitopes. **(B)** Spatial localization of the epitopes identified by the microarray (M50) (left) and AbMap (right) analysis on the trimer model of the S protein (side view). The backbone structure is illustrated in the gray cartoon mode. Each set of epitopes are highlighted on the surface. Epitopes located in the S1, S2, and transmembrane regions are shown in yellow, light blue, and dark blue, respectively. **(C)** Spatial localization of the five S epitopes with α-helical structures on the trimer model of S2 protein. Stalk region of the S2 protein is shown in gray cartoon. The five peptide epitopes, namely, S2-15, S2-45, S2-56, S2-78, and S2-83 are shown in purple, yellow, green, cyan, and blue respectively.

The secondary structures of the S peptides that were designed for microarray analysis were analyzed using DSSP, and the prediction results were illustrated in **Supplementary Table 3**. Peptides negatively recognized by the COVID-19 sera showed a significantly higher percentage of alpha helical structures and a significantly lower percentage of β-sheet and random coil structures than those that reacted positively with the patient serum samples (alpha-helix: 23.2% vs. 18.7%; β-sheet: 21.4% vs. 23.1% and random coil: 51.4% vs. 58.2%). Furthermore, the alpha helix percentage decreased to 17.7% and the random coil percentage increased to 59.6% in the positive S peptides with 50% frequency in the COVID-19 patients (M50) (**Figure 5A**). The S epitopes corresponding to the matched motifs by AbMap displayed a similar distribution of secondary structures (13.4% alpha helix and 65% random coils; **Figure 5A**). These data suggested that S peptides with higher β-sheets or random coil secondary structures were easily recognized by the humoral immune system. This conclusion agrees with epitope theory that random coils possess a higher potential for antigenicity (39, 40).

The cryo-EM model of the trimeric S protein demonstrated that the four domains of S1, namely NTD, RBD, CTD1, and CTD2, wrap around a threefold axis and cover S2; moreover, the surface-exposed and disordered loop model showed the furin cleavage site at the S1/S2 boundary (41). To overview the spatial structures of the S epitopes, Pymol was applied to map the identified S epitopes onto the molecular model of the S protein in the closed state. The M50 peptides were mainly located in the surface-exposed regions of S1 (25/40; **Figure 5B**). The spatial locations of the S epitopes corresponding to the matched motifs by AbMap were also mapped to the three-dimensional model of S proteins. Approximately 62% of the epitopes were exposed on the S protein surface (**Figure 5B**). Therefore, the immunogenicity of the S protein is well explained by the location of immune-positive peptides in the tertiary structure of the S protein. Moreover, the relatively poorer antigenicity of S2 may be related to its higher percentage of alpha helices (40%) compared to the low percentage of alpha helical structures in S1 in the closed state.

Among the M50 S peptides, five peptides with a higher helical content (>75%) were well-recognized by COVID-19 sera. In the spatial structure of the S protein, three peptides (S2-15, S2-45 and S2-56) were located around the FP region of S2 and shielded by the CTD2 region of S1 in the closed trimer, whereas two peptides (S2-78 and S2-83) were located in the HR2 region of S2, which is close to the viral membrane (**Figure 5C**). Closely checking the status of the three former peptides in response to viral infection, S protein is likely cleaved by furin-like protease followed by cleavage of serine protease; then, the truncated S2 proteins would be expected to be in an exposure status and bring some configuration changes (42), which influence their antigenicity and recognition by the humoral immune system. With regard to the two later peptides, these HR2 peptides are anticipated to be in an exposed location upon the tertiary structure of the S protein. In addition, the S epitopes derived from AbMap also supported the above deduction; epitopes (epi-23: residues 771–778 and epi-25: residues 1,019–1,028) overlapped with S2–15 (residues 770–781) and S2–56 (residues 1016–1,027), respectively.

## Discussion

Considering the technology bias of misjudging epitope recognition, in this study, three types of S antigens, recombinant truncated S proteins, S peptides, and random peptides, were used to examine the dynamics of humoral immune responses in the sera of COVID-19 patients. The question naturally arises as to how experimental evidence supports the theoretical design. First of all, the patterns of longitudinal reactivity of the patient IgM and IgG against these antigens, intact S protein measured by ELISA, recombinant S1 protein and commonly recognized S peptides detected by microarray, were similar, with the IgM responses rising early and IgG coming later. Secondly, the distribution of the positive S peptides with higher frequencies identified by microarray along the *S* gene was similar to the regions of the S epitopes found by AbMap. Thus, the two approaches to explore the S epitopes recognized by the COVID-19 sera reached an agreed conclusion. Finally, both the S peptides microarray and AbMap results showed that the recognition of the S epitopes by IgG varied among different individuals, but the humoral immune responses against certain the S epitopes were relatively stable in individual COVID-19 patient for a period of two months after symptom onset. Hence, the main conclusions regarding the S epitopes recognized by humoral immune responses were well endorsed by multiple datasets obtained from different approaches.

Several studies have monitored the humoral immune responses to SARS-CoV-2 infection and to identify viral antigens through serological assays (23, 43, 44). In the present study, ELISA demonstrated that the IgM reactivity peak was obtained during the first two weeks after symptom onset, whereas the IgG reactivity peak was observed around the fifth or sixth week after symptom onset in the COVID-19 cohort (**Figure 1A**). These observations are consistent with those of previous studies (22, 45, 46). The evidence obtained from the microarray with truncated S proteins as antigens supported our previous conclusion that the intensity of immune responses against S1 was significantly higher than that against S2 and RBD domains of the S protein (35). However, the humoral immune responses against viral antigens such as S1, S2, and RBD of the S protein are still contradictory (21, 47–51). Premkumar et al. reported that RBD is immunodominant and a highly specific target for humoral immune system in COVID-19 patients (51). Nguyen et al. compared the antigenicity of S, S1, S2, and RBD by ELISA and reported that S2 and S proteins were preferentially recognized by patient antibodies at two weeks after symptom onset (50). Norman et al. performed an ultra-sensitive single molecular array (Simoa) assay and reported similar binding capacities for IgA, IgM, and IgG antibodies against S1, RBD, and S protein in patients with COVID-19 (48). However, Tian et al. demonstrated that S1 displayed higher sensitivity and specificity than RBD (52). With solid data support from three approaches, the conclusion elicited from this study advocated that the antigenicity of S1 was higher than that of S2 and RBD.

As mentioned above, 124 IgM- and 165 IgG-reactive S peptides were identified through serological assays using the sera of COVID-19 patients. To extract S peptides commonly recognized by individual patients, a new concept M50 was introduced in this study. The epitope distribution along the *S* gene and epitope accessibility of the S protein is well elucidated by the M50 peptides. Recently, several SARS-COV-2 variants have been reported, especially several variants of concern (VOCs). All the M50 S peptides were compared with the varied sequences of S protein in VOCs (CoV-GLUE-Viz), while the comparison revealed 30% (12/40) of M50 S peptides containing the mutated amino acid residues, indicating that the variants of SARS-COV-2 are likely to affect humoral immune responses against the virus (**Supplementary Table 4**). Specifically, for the epitope identified in M50, seven peptides (S1-1, S1-3, S1-33, S1-35, S1-101, S1-105 and S2-78) were generally recognized by IgG of all the patients with COVID-19 in this cohort. In addition, the panel with the seven S peptides showed dynamic patterns similar to those of the S1 protein. The four S peptides in this panel were defined in previous reports as S1-35 (residues 205–216) in NTD (53), S1-101 (residues 601–612) and S1-105 (residues 635–636) in CTD (54), and S2-78 (residues 1,148–1,159) in S2 adjacent to HR2 (54–57). The remaining three peptide epitopes, S1-1 (residues 1–12), S1-3 (residues 13–24) and S1-33 (residues 193–204) were first identified by this study. The clinical value of these general and new epitopes will be verified and explored in future studies.

The human immune system is highly variable between individuals but relatively stable over time within a given person (58). Xiang et al. studied the B-cell immune repertoire of COVID-19 patients and reported that despite significant differences in *V* gene usage among the COVID-19 patients, the frequency of different *V* and *J* gene segment usage remained relatively stable over time in individual COVID-19 patients (34). Niu et al. reported that the IgM and IgG expression in B cells at transcript levels displayed a large diversity at the early SARS-CoV-2 infection within four days, whereas the diversity in the continued clonal expansion of dominant B cells decreased after recovery from infection (32). Nevertheless, details regarding the dynamic nature of epitope recognition during the course of SARS-CoV-2 infection are unclear. Therefore, we systematically assessed the dynamic humoral immune response against the S protein or S peptides. Our results showed that the recognition of S epitopes by IgG and IgM antibodies was highly diverse and patient-specific. However, the pattern of recognizing certain general or individual-specific S epitopes by IgM or IgG antibodies was consistent in each patient with COVID-19 during the hospitalization period (**Figures 3A, B**, **4C**). The observation suggested that B cells undergo a series of transcriptional edits in response to SARS-CoV-2 infection during the early phase of infection, while the specific clones are selected and the IgM and IgG antibodies matured during the period of infection. Thus, after immunoglobins against the viral antigens are mature their recognition affinities to certain S epitopes in a given individual are almost fixed to provide effective humoral immunity for a long duration after symptom onset. The longitudinal characterization of humoral immunity to SARS-COV-2 may contribute novel information on how to consider a proper therapy for COVID-19 patients, especially during the early phase of infection.

## Data Availability Statement

The original contributions presented in the study are included in the article/**Supplementary Material**. Further inquiries can be directed to the corresponding author.

## Ethics Statement

The studies involving human participants were reviewed and approved by the Research Ethics Committee in the Fifth Affiliated Hospital of Sun Yat-sen University. The patients/participants provided their written informed consent to participate in this study.

## Author Contributions

SL, ST, and HS conceived of the study and prepared the manuscript. LC designed the experiments, analyzed the data, and assisted with manuscript preparation. HQ and MM performed epitope mapping of protein-enriched antibodies. YL, HZ HJ, and DL performed microarray experiments. JX, HL, and CH performed NGS and data analysis. KY analyzed the data and prepared the figures. RC and YL provided suggestions for the spatial analysis of the epitopes. PP and YR collected the clinical samples and acquired the data. All authors listed have made a substantial, direct, and intellectual contribution to the work and approved it for publication.

## Funding

This study was supported by the National Key Research and Development Program of China (Grant Nos. 2016YFA0500600 and 2018YFC1003100) and the National Natural Science Foundation of China (Grant Nos. 31970130, 31600672, 31900112, 21907065, 32000027, and 81873478).

## Conflict of Interest

Authors LC, KY, YR, RC, YD and SL are employed by Beijing Genomics Institution.

The remaining authors declare that the research was conducted in the absence of any commercial or financial relationships that could be construed as a potential conflict of interest.

## Publisher’s Note

All claims expressed in this article are solely those of the authors and do not necessarily represent those of their affiliated organizations, or those of the publisher, the editors and the reviewers. Any product that may be evaluated in this article, or claim that may be made by its manufacturer, is not guaranteed or endorsed by the publisher.
