# Supplementary material for: Evaluation of Spike Protein Epitopes by Assessing the Dynamics of Humoral Immune Responses in Moderate COVID-19
Source: Front Immunol. 2022 Mar 18;13:770982. doi: 10.3389/fimmu.2022.770982 (PMC8971992; doi:10.3389/fimmu.2022.770982)
Supplement: Supplementary file 1 [file DataSheet_1.pdf]

## *Supplementary Material*

### Supplementary Figures and Tables

### Supplementary Figures

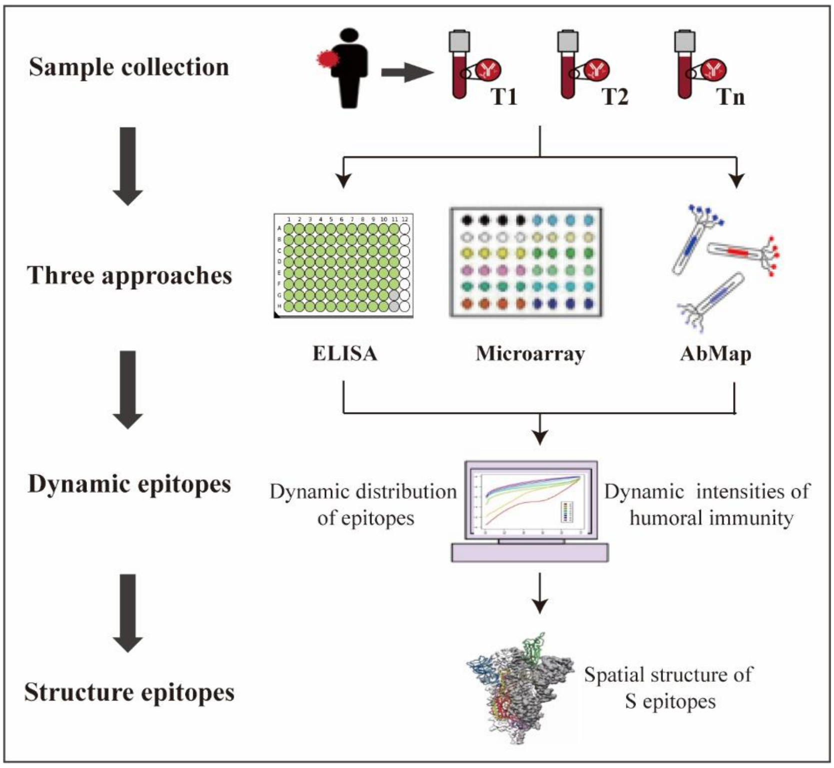


**Supplementary Figure 1 Schematic of experiment design and data analysis.**

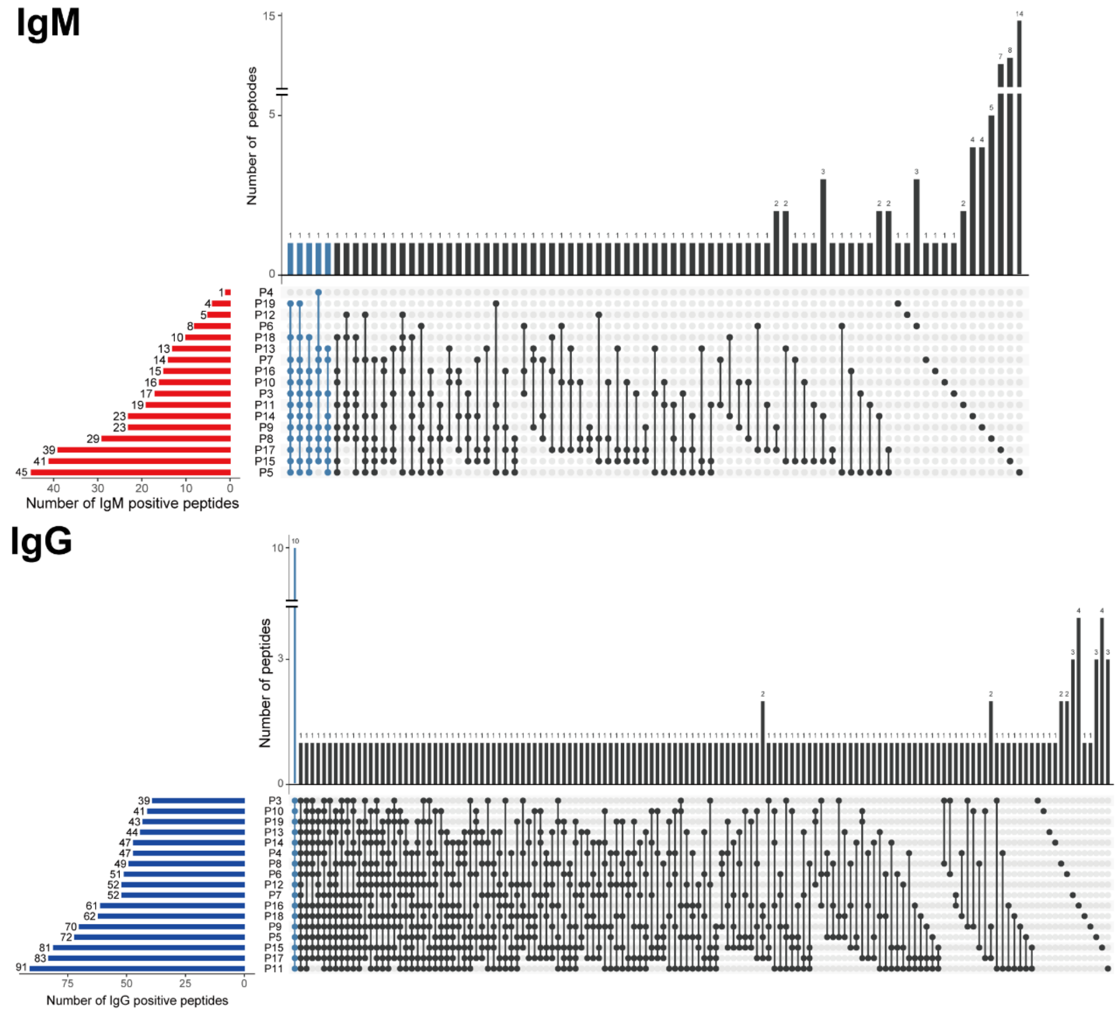


**Supplementary Figure 2 Distribution of the S peptides positively recognized by IgM and IgG antibodies of the COVID-19 patients.** On the left, the histograms indicate the total number of IgM-reactive or IgG-reactive S peptides identified in each COVID-19 patient. On the right, the blue and black bars represent the number of S peptides with high and low frequencies in this cohort, for IgM the higher frequency by set at ≥50% and the lower at <50%, and for IgG the higher frequency at 100% and the lower at <100%. The blue and black dots corresponding to the patients (Y axis) denote whether a S peptide is recognized by a patient serum.

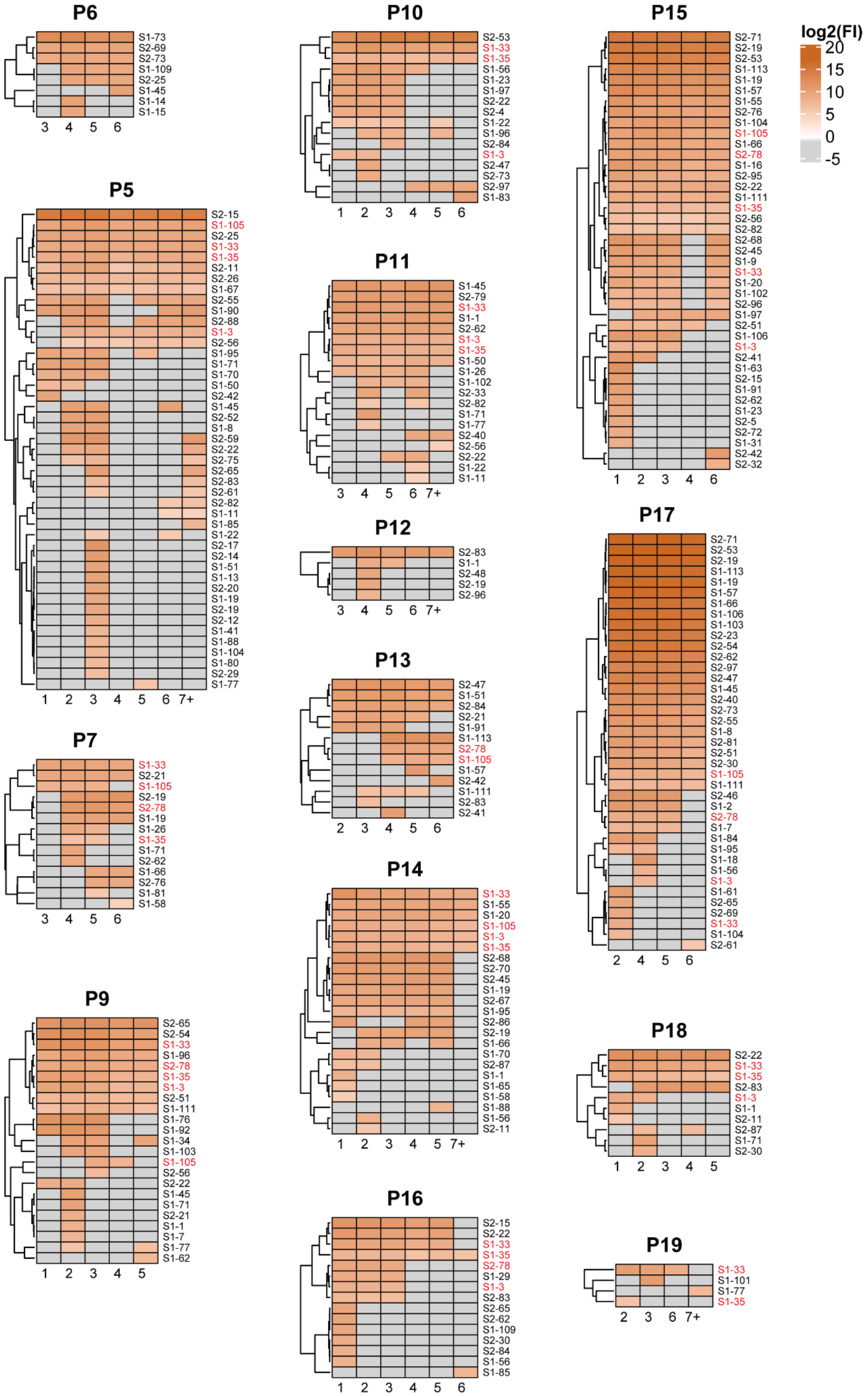
P6
P10
P15
log2(FI)
P5
P11
P12
P13
P7
P14
P17
P9
P18
P16
P19

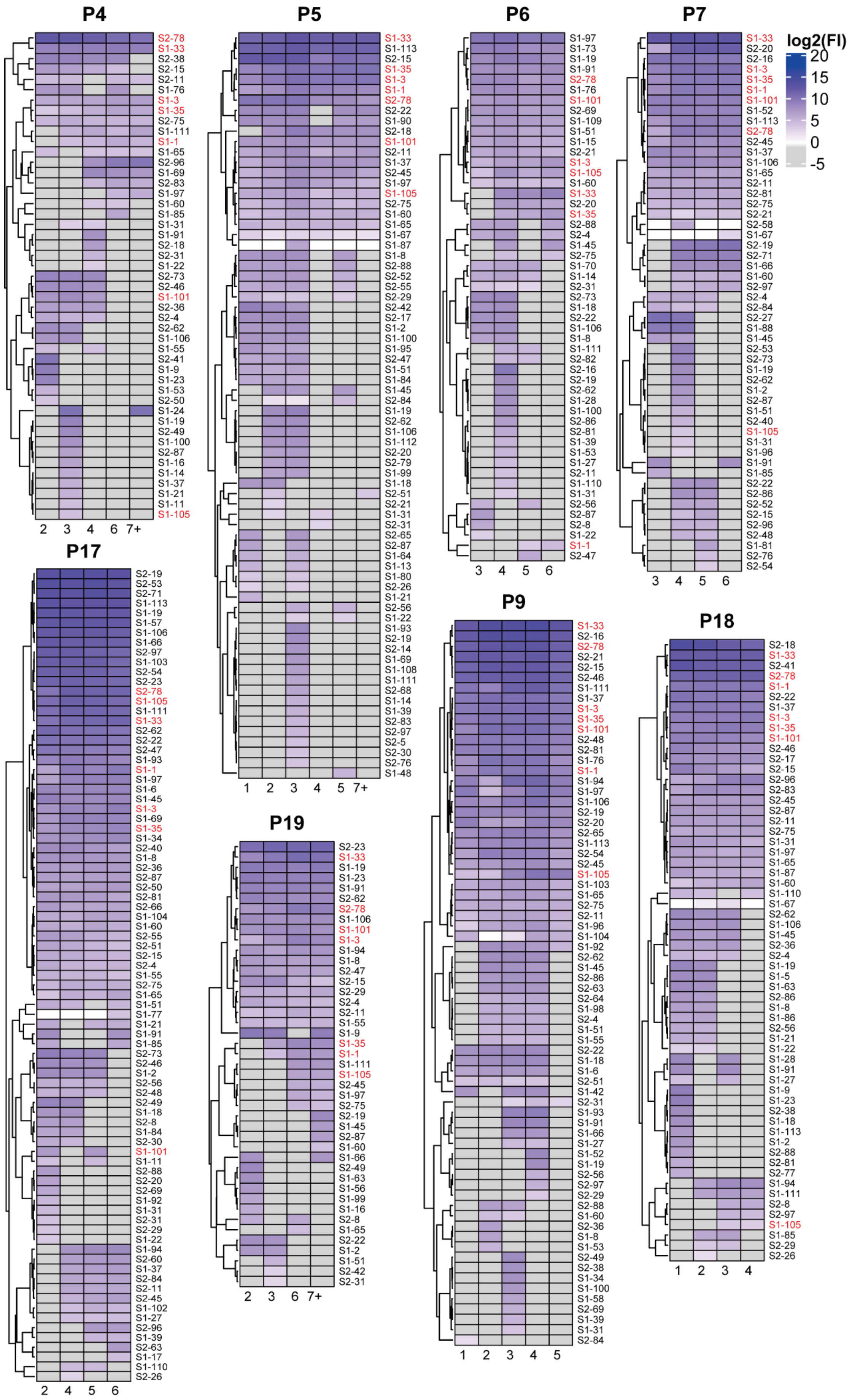
P4
S2-78
S1-33
S2-38
S2-15
S2-11
S1-76
S1-3
S1-35
S2-75
S1-111
S1-1
S1-65
S2-96
S1-69
S2-83
S1-97
S1-60
S1-85
S1-31
S1-91
S2-18
S2-31
S1-22
S2-73
S2-46
S1-101
S2-36
S2-4
S2-62
S1-106
S1-55
S2-41
S1-9
S1-23
S1-53
S2-50
S1-24
S1-19
S2-49
S1-100
S2-87
S1-16
S1-14
S1-37
S1-21
S1-11
S1-105
2 3 4 6 7+
P5
S1-33
S1-113
S2-15
S1-35
S1-3
S1-1
S2-78
S2-22
S1-90
S2-18
S1-101
S2-11
S1-37
S2-45
S1-97
S1-105
S2-75
S1-60
S1-65
S1-67
S1-87
S1-8
S2-88
S2-52
S2-55
S2-29
S2-42
S2-17
S1-2
S1-100
S1-95
S2-47
S1-51
S1-84
S1-45
S2-84
S1-19
S2-62
S1-106
S1-112
S2-20
S2-79
S1-99
S1-18
S2-51
S2-21
S1-31
S2-31
S2-65
S2-87
S1-64
S1-13
S1-80
S2-26
S1-21
S2-56
S1-22
S1-93
S2-19
S2-14
S1-69
S1-108
S1-111
S2-68
S1-14
S1-39
S2-83
S2-97
S2-5
S2-30
S2-76
S1-48
1 2 3 4 5 7+
P6
S1-97
S1-73
S1-19
S1-91
S2-78
S1-76
S1-101
S2-69
S1-109
S1-51
S1-15
S2-21
S1-3
S1-105
S1-60
S1-33
S2-20
S1-35
S2-88
S2-4
S1-45
S2-75
S1-70
S1-14
S2-31
S2-73
S1-18
S2-22
S1-106
S1-8
S1-111
S2-82
S2-16
S2-19
S2-62
S1-28
S1-100
S2-86
S2-81
S1-39
S1-53
S1-27
S2-11
S1-110
S1-31
S2-56
S2-87
S2-8
S1-22
S1-1
S2-47
3 4 5 6
P7
S1-33
S2-20
S2-16
S1-3
S1-35
S1-1
S1-101
S1-52
S1-113
S2-78
S2-45
S1-37
S1-106
S1-65
S2-11
S2-81
S2-75
S2-21
S2-58
S1-67
S2-19
S2-71
S1-66
S1-60
S2-97
S2-4
S2-84
S2-27
S1-88
S1-45
S2-53
S2-73
S1-19
S2-62
S1-2
S2-87
S1-51
S2-40
S1-105
S1-31
S1-96
S1-91
S1-85
S2-22
S2-86
S2-52
S2-15
S2-96
S2-48
S1-81
S2-76
S2-54
3 4 5 6
log2(FI)
20
15
10
5
0
-5
P17
S2-19
S2-53
S2-71
S1-113
S1-19
S1-57
S1-106
S1-66
S2-97
S1-103
S2-54
S2-23
S2-78
S1-105
S1-111
S1-33
S2-62
S2-22
S2-47
S1-93
S1-1
S1-97
S1-6
S1-45
S1-3
S1-69
S1-35
S1-34
S2-40
S1-8
S2-36
S2-87
S2-50
S2-81
S2-66
S1-104
S1-60
S2-55
S2-51
S2-15
S2-4
S1-55
S2-75
S1-65
S1-51
S1-77
S1-21
S1-91
S1-85
S2-73
S2-46
S1-2
S2-56
S2-48
S2-49
S1-18
S2-8
S1-84
S2-30
S1-101
S1-11
S2-88
S2-20
S2-69
S1-92
S1-31
S2-31
S2-29
S1-22
S1-94
S2-60
S1-37
S2-84
S2-11
S2-45
S1-102
S1-27
S2-96
S1-39
S2-63
S1-17
S1-110
S2-26
2 4 5 6
P19
S2-23
S1-33
S1-19
S1-23
S1-91
S2-62
S2-78
S1-106
S1-101
S1-3
S1-94
S1-8
S2-47
S2-15
S2-29
S2-4
S2-11
S1-55
S1-9
S1-35
S1-1
S1-111
S1-105
S2-45
S1-97
S2-75
S2-19
S1-45
S2-87
S1-60
S1-66
S2-49
S1-63
S1-56
S1-99
S1-16
S2-8
S1-65
S2-22
S1-2
S1-51
S2-42
S2-31
2 3 6 7+
P9
S1-33
S2-16
S2-78
S2-21
S2-15
S2-46
S1-111
S1-37
S1-3
S1-35
S1-101
S2-48
S2-81
S1-76
S1-1
S1-94
S1-97
S1-106
S2-19
S2-20
S2-65
S1-113
S2-54
S2-45
S1-105
S1-103
S1-65
S2-75
S2-11
S1-96
S1-104
S1-92
S2-62
S1-45
S2-86
S2-63
S2-64
S1-98
S2-4
S1-51
S1-55
S2-22
S1-18
S1-6
S2-51
S1-42
S2-31
S1-93
S1-91
S1-66
S1-27
S1-52
S1-19
S2-56
S2-97
S2-29
S2-88
S1-60
S2-36
S1-8
S1-53
S2-49
S2-38
S1-34
S1-100
S1-58
S2-69
S1-39
S1-31
S2-84
1 2 3 4 5
P18
S2-18
S1-33
S2-41
S2-78
S1-1
S2-22
S1-37
S1-3
S1-35
S1-101
S2-46
S2-17
S2-15
S2-96
S2-83
S2-45
S2-87
S2-11
S2-75
S1-31
S1-97
S1-65
S1-87
S1-60
S1-110
S1-67
S2-62
S1-106
S1-45
S2-36
S2-4
S1-19
S1-5
S1-63
S2-86
S1-8
S1-86
S2-56
S1-21
S1-22
S1-28
S1-91
S1-27
S1-9
S1-23
S2-38
S1-18
S1-113
S1-2
S2-88
S2-81
S2-77
S1-94
S1-111
S2-8
S2-97
S1-105
S1-85
S2-29
S2-26
1 2 3 4

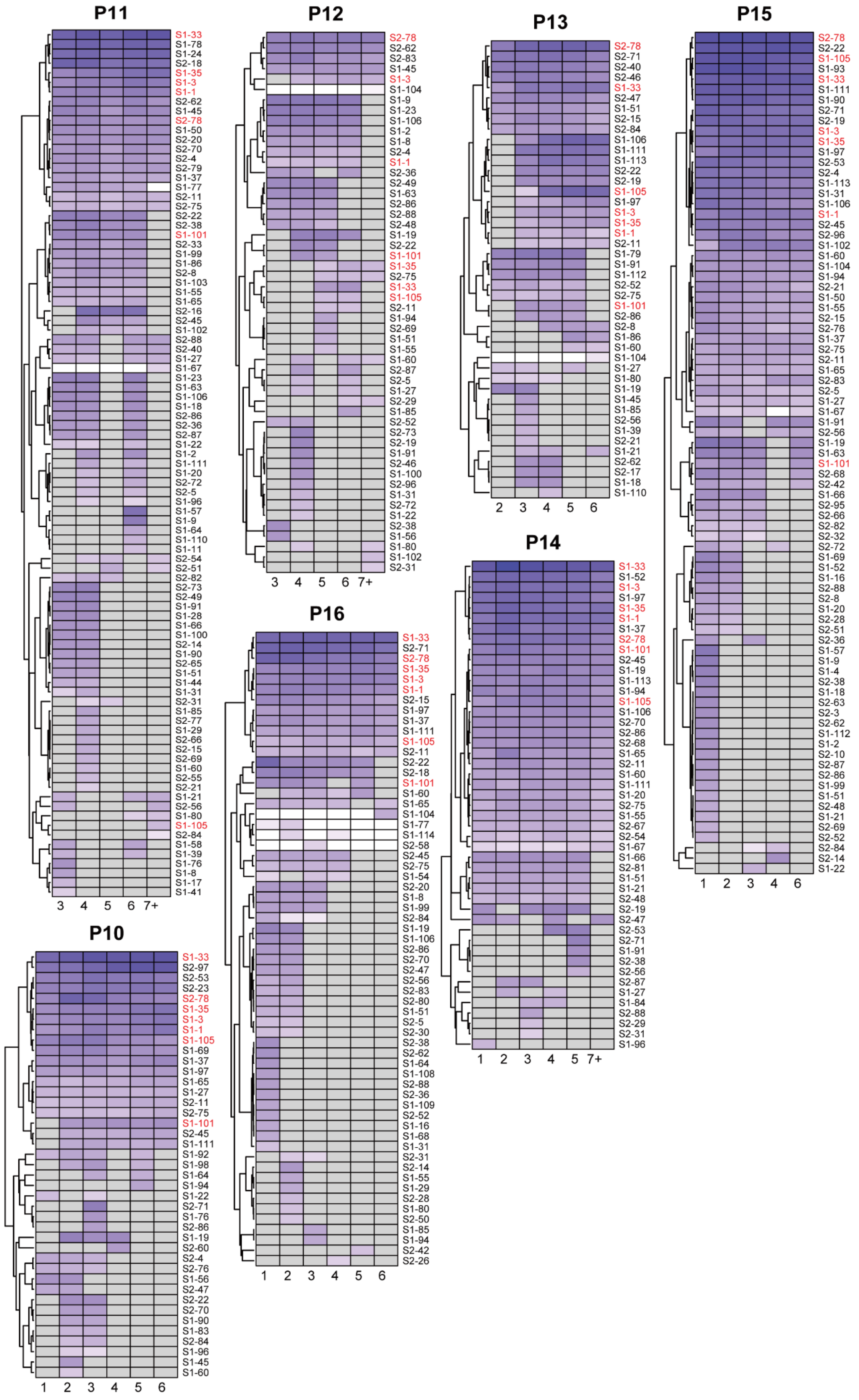
P11
S1-33
S1-78
S1-24
S2-18
S1-35
S1-3
S1-1
S2-62
S1-45
S2-78
S1-50
S2-20
S2-70
S2-4
S2-79
S1-37
S1-77
S2-11
S2-75
S2-22
S2-38
S1-101
S2-33
S1-99
S1-86
S2-8
S1-103
S1-55
S1-65
S2-16
S2-45
S1-102
S2-88
S2-40
S1-27
S1-67
S1-23
S1-63
S1-106
S1-18
S2-86
S2-36
S2-87
S1-22
S1-2
S1-111
S1-20
S2-72
S2-5
S1-96
S1-57
S1-9
S1-64
S1-110
S1-11
S2-54
S2-51
S2-82
S2-73
S2-49
S1-91
S1-28
S1-66
S1-100
S2-14
S1-90
S2-65
S1-51
S1-44
S1-31
S2-31
S1-85
S2-77
S1-29
S2-66
S2-15
S2-69
S1-60
S2-55
S2-21
S1-21
S2-56
S1-80
S1-105
S2-84
S1-58
S1-39
S1-76
S1-8
S1-17
S1-41
3 4 5 6 7+
P12
S2-78
S2-62
S2-83
S1-45
S1-3
S1-104
S1-9
S1-23
S1-106
S1-2
S1-8
S2-4
S1-1
S2-36
S2-49
S1-63
S2-86
S2-88
S2-48
S1-19
S2-22
S1-101
S1-35
S2-75
S1-33
S1-105
S2-11
S1-94
S2-69
S1-51
S1-55
S1-60
S2-87
S2-5
S1-27
S2-29
S1-85
S2-52
S2-73
S2-19
S1-91
S2-46
S1-100
S2-96
S1-31
S2-72
S1-22
S2-38
S1-56
S1-80
S1-102
S2-31
3 4 5 6 7+
P13
S2-78
S2-71
S2-40
S2-46
S1-33
S2-47
S1-51
S2-15
S2-84
S1-106
S1-111
S1-113
S2-22
S2-19
S1-105
S1-97
S1-3
S1-35
S1-1
S2-11
S1-79
S1-91
S1-112
S2-52
S2-75
S1-101
S2-86
S2-8
S1-86
S1-60
S1-104
S1-27
S1-80
S1-19
S1-45
S1-85
S2-56
S1-39
S2-21
S1-21
S2-62
S2-17
S1-18
S1-110
2 3 4 5 6
P15
S2-78
S2-22
S1-105
S1-93
S1-33
S1-111
S1-90
S2-71
S2-19
S1-3
S1-35
S1-97
S2-53
S2-4
S1-113
S1-31
S1-106
S1-1
S2-45
S2-96
S1-102
S1-60
S1-104
S1-94
S2-21
S1-50
S1-55
S2-15
S2-76
S1-37
S2-75
S2-11
S1-65
S2-83
S2-5
S1-27
S1-67
S1-91
S2-56
S1-19
S1-63
S1-101
S2-68
S2-42
S1-66
S2-95
S2-66
S2-82
S2-32
S2-72
S1-69
S1-52
S1-16
S2-88
S2-8
S1-20
S2-28
S2-51
S2-36
S1-57
S1-9
S1-4
S2-38
S1-18
S2-63
S2-3
S2-62
S1-112
S1-2
S2-10
S2-87
S2-86
S1-99
S1-51
S2-48
S1-21
S2-69
S2-52
S2-84
S2-14
S1-22
1 2 3 4 6
P14
S1-33
S1-52
S1-3
S1-97
S1-35
S1-1
S1-37
S2-78
S1-101
S2-45
S1-19
S1-113
S1-94
S1-105
S1-106
S2-70
S2-86
S2-68
S1-65
S2-11
S1-60
S1-111
S1-20
S2-75
S1-55
S2-67
S2-54
S1-67
S1-66
S2-81
S1-51
S1-21
S2-48
S2-19
S2-47
S2-53
S2-71
S1-91
S2-38
S2-56
S2-87
S1-27
S1-84
S2-88
S2-29
S2-31
S1-96
1 2 3 4 5 7+
P16
S1-33
S2-71
S2-78
S1-35
S1-3
S1-1
S2-15
S1-97
S1-37
S1-111
S1-105
S2-11
S2-22
S2-18
S1-101
S1-60
S1-65
S1-104
S1-77
S1-114
S2-58
S2-45
S2-75
S1-54
S2-20
S1-8
S1-99
S2-84
S1-19
S1-106
S2-86
S2-70
S2-47
S2-56
S2-83
S2-80
S1-51
S2-5
S2-30
S2-38
S2-62
S1-64
S1-108
S2-88
S2-36
S1-109
S2-52
S1-16
S1-68
S1-31
S2-31
S2-14
S1-55
S1-29
S2-28
S1-80
S2-50
S1-85
S1-94
S2-42
S2-26
1 2 3 4 5 6
P10
S1-33
S2-97
S2-53
S2-23
S2-78
S1-35
S1-3
S1-1
S1-105
S1-69
S1-37
S1-97
S1-65
S1-27
S2-11
S2-75
S1-101
S2-45
S1-111
S1-92
S1-98
S1-64
S1-94
S1-22
S2-71
S1-76
S2-86
S1-19
S2-60
S2-4
S2-76
S1-56
S2-47
S2-22
S2-70
S1-90
S1-83
S2-84
S1-96
S1-45
S1-60
1 2 3 4 5 6

**Supplementary Figure 3** Heatmaps of dynamics IgM (the cells colored in brown indicate positive signals) and IgG (the cells colored in blue indicate positive signals) against the S-reactive peptides in individual COVID-19 patient during hospitalization after symptom onset. The gray cells indicate the corresponding immune signals with values lower than the cutoffs. The gradient bar with brown or blue indicates the intensities of immune reactions for IgM or IgG. The x axis of heatmap indicates the time intervals (week) during the hospitalization after symptom onset.

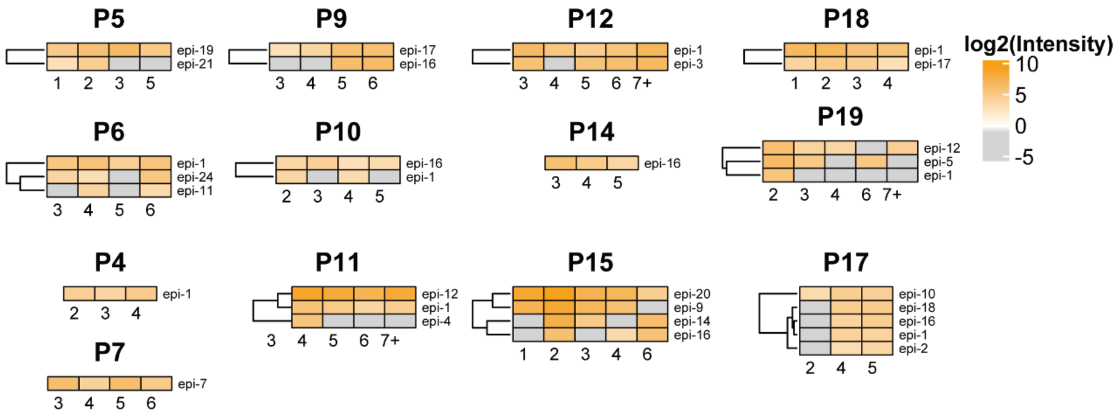


**Supplementary Figure 4** Heatmaps of the S epitopes recognized by the purified IgG in the individual COVID-19 patients. The gray cells indicate unmatched S epitopes identified from the corresponding samples. The gradient bar with orange indicates the intensities of immune reactions for IgG against S epitopes. The x axis of heatmaps indicate the time intervals (week) during the hospitalization after symptom onset.

## Supplementary Tables

**Supplementary Table 1** Time of blood collection was based on the number of days after symptom onset.

| Patient | The time for collecting blood samples (Days after onset of the symptoms) | | | | | | | | | | |
|---|---|---|---|---|---|---|---|---|---|---|---|
| P1 | 17 | 19 | 32 | | | | | | | | |
| P2 | 18 | 23 | | | | | | | | | |
| P3 | 4 | 6 | 8 | 11 | 16 | 30 | 37 | | | | |
| P4 | 13 | 15 | 18 | 22 | 24 | 38 | 45 | | | | |
| P5 | 6 | 8 | 11 | 15 | 23 | 30 | 36 | 48 | | | |
| P6 | 19 | 21 | 24 | 28 | 35 | 37 | 42 | | | | |
| P7 | 19 | 21 | 24 | 28 | 35 | 42 | | | | | |
| P8 | 13 | 15 | 31 | 38 | 54 | | | | | | |
| P9 | 5 | 13 | 15 | 18 | 22 | 31 | | | | | |
| P10 | 7 | 13 | 16 | 20 | 26 | 33 | 40 | | | | |
| P11 | 18 | 20 | 23 | 27 | 35 | 42 | 48 | | | | |
| P12 | 19 | 21 | 24 | 28 | 33 | 40 | 47 | 61 | | | |
| P13 | 14 | 16 | 18 | 19 | 23 | 30 | 37 | | | | |

| | | | | | | | | | | |
|---|---|---|---|---|---|---|---|---|---|---|
| P14 | 7 | 13 | 18 | 21 | 27 | 33 | 45 | | | |
| P15 | 4 | 8 | 10 | 11 | 13 | 15 | 27 | 41 | | |
| P16 | 2 | 4 | 6 | 8 | 10 | 13 | 15 | 18 | 23 | 29 | 41 |
| P17 | 13 | 24 | 31 | 38 | | | | | | | |
| P18 | 4 | 8 | 10 | 12 | 16 | 23 | 30 | | | | |
| P19 | 10 | 12 | 15 | 19 | 37 | 44 | | | | | |

**Supplementary Table 2** Cases of the blood samples and patients with the COVID-19 patients were based on weeks after symptom onset.

| | Week1 | Week2 | Week3 | Week4 | Week5 | Week6 | Week7/+ |
|---|---|---|---|---|---|---|---|
| **Blood samples** | 11 | 23 | 31 | 20 | 15 | 15 | 8 |
| **Patients** | 8 | 13 | 18 | 15 | 15 | 17 | 7 |

**Supplementary Table 3** Positive frequency recognized by COVID-19 patients and the percentiles of the secondary structure of all S peptides. Forty IgG-reactive S peptides with 50% frequency in the COVID-19 patients (M50) are labeled with star.

| Peptide ID | Position | Positive Frequency | α-helix | β-sheet | Coil | Exposed percent |
|---|---|---|---|---|---|---|
| *S1-1 | [1, 12] | 1.000 | 0.000 | 0.000 | 1.000 | 0.833 |
| S1-2 | [7, 18] | 0.471 | 0.000 | 0.000 | 1.000 | 0.833 |
| *S1-3 | [13, 24] | 1.000 | 0.000 | 0.000 | 1.000 | 0.833 |
| S1-4 | [19, 30] | 0.059 | 0.000 | 0.333 | 0.667 | 0.833 |
| S1-5 | [25, 36] | 0.059 | 0.000 | 0.417 | 0.583 | 0.667 |
| S1-6 | [31, 42] | 0.118 | 0.000 | 0.250 | 0.750 | 0.417 |
| S1-7 | [37, 48] | 0.000 | 0.000 | 0.250 | 0.750 | 0.583 |
| *S1-8 | [43, 54] | 0.588 | 0.000 | 0.333 | 0.667 | 0.750 |
| S1-9 | [49, 60] | 0.353 | 0.000 | 0.333 | 0.667 | 0.500 |
| S1-10 | [55, 66] | 0.000 | 0.000 | 0.583 | 0.417 | 0.417 |
| S1-11 | [61, 72] | 0.176 | 0.000 | 0.500 | 0.500 | 0.667 |
| S1-12 | [67, 78] | 0.000 | 0.000 | 0.000 | 1.000 | 0.583 |
| S1-13 | [73, 84] | 0.059 | 0.000 | 0.083 | 0.917 | 0.417 |
| S1-14 | [79, 90] | 0.176 | 0.000 | 0.333 | 0.667 | 0.417 |
| S1-15 | [85, 96] | 0.059 | 0.000 | 0.750 | 0.250 | 0.250 |
| S1-16 | [91, 102] | 0.235 | 0.000 | 0.667 | 0.333 | 0.083 |
| S1-17 | [97, 108] | 0.176 | 0.000 | 0.583 | 0.417 | 0.167 |
| *S1-18 | [103, 114] | 0.529 | 0.000 | 0.417 | 0.583 | 0.444 |
| *S1-19 | [109, 120] | 0.824 | 0.000 | 0.417 | 0.583 | 0.361 |
| S1-20 | [115, 126] | 0.176 | 0.000 | 0.417 | 0.583 | 0.417 |
| *S1-21 | [121, 132] | 0.529 | 0.000 | 0.083 | 0.917 | 0.583 |
| *S1-22 | [127, 138] | 0.588 | 0.000 | 0.167 | 0.833 | 0.250 |
| S1-23 | [133, 144] | 0.294 | 0.000 | 0.250 | 0.750 | 0.167 |

| | | | | | | |
|---|---|---|---|---|---|---|
| S1-24 | [139, 150] | 0.118 | 0.000 | 0.250 | 0.750 | 0.500 |
| S1-25 | [145, 156] | 0.000 | 0.000 | 0.167 | 0.833 | 0.750 |
| S1-26 | [151, 162] | 0.000 | 0.000 | 0.167 | 0.833 | 0.750 |
| *S1-27 | [157, 168] | 0.706 | 0.000 | 0.167 | 0.833 | 0.917 |
| S1-28 | [163, 174] | 0.176 | 0.000 | 0.083 | 0.917 | 1.000 |
| S1-29 | [169, 180] | 0.118 | 0.000 | 0.000 | 1.000 | 1.000 |
| S1-30 | [175, 186] | 0.000 | 0.000 | 0.000 | 1.000 | 0.917 |
| *S1-31 | [181, 192] | 0.647 | 0.000 | 0.417 | 0.583 | 0.583 |
| S1-32 | [187, 198] | 0.000 | 0.000 | 0.833 | 0.167 | 0.417 |
| *S1-33 | [193, 204] | 1.000 | 0.000 | 0.833 | 0.167 | 0.444 |
| S1-34 | [199, 210] | 0.118 | 0.000 | 0.750 | 0.250 | 0.556 |
| *S1-35 | [205, 216] | 1.000 | 0.000 | 0.333 | 0.667 | 0.778 |
| S1-36 | [211, 222] | 0.000 | 0.000 | 0.083 | 0.917 | 0.833 |
| *S1-37 | [217, 228] | 0.765 | 0.000 | 0.583 | 0.417 | 0.833 |
| S1-38 | [223, 234] | 0.059 | 0.000 | 0.667 | 0.333 | 0.667 |
| S1-39 | [229, 240] | 0.353 | 0.000 | 0.500 | 0.500 | 0.417 |
| S1-40 | [235, 246] | 0.000 | 0.000 | 0.583 | 0.417 | 0.250 |
| S1-41 | [241, 252] | 0.118 | 0.000 | 0.250 | 0.750 | 0.472 |
| S1-42 | [247, 258] | 0.059 | 0.000 | 0.000 | 1.000 | 0.806 |
| S1-43 | [253, 264] | 0.000 | 0.000 | 0.083 | 0.917 | 0.528 |
| S1-44 | [259, 270] | 0.059 | 0.000 | 0.500 | 0.500 | 0.194 |
| *S1-45 | [265, 276] | 0.647 | 0.000 | 0.750 | 0.250 | 0.417 |
| S1-46 | [271, 282] | 0.000 | 0.000 | 0.583 | 0.417 | 0.667 |
| S1-47 | [277, 288] | 0.000 | 0.000 | 0.583 | 0.417 | 0.667 |
| S1-48 | [283, 294] | 0.059 | 0.000 | 0.500 | 0.500 | 0.583 |
| S1-49 | [289, 300] | 0.000 | 0.500 | 0.167 | 0.333 | 0.500 |
| S1-50 | [295, 306] | 0.118 | 0.500 | 0.000 | 0.500 | 0.667 |
| *S1-51 | [301, 312] | 0.824 | 0.000 | 0.167 | 0.833 | 0.833 |
| S1-52 | [307, 318] | 0.235 | 0.000 | 0.417 | 0.583 | 0.833 |
| S1-53 | [313, 324] | 0.235 | 0.000 | 0.333 | 0.667 | 0.917 |
| S1-54 | [319, 330] | 0.059 | 0.000 | 0.250 | 0.750 | 0.750 |
| *S1-55 | [325, 336] | 0.588 | 0.000 | 0.333 | 0.667 | 0.750 |
| S1-56 | [331, 342] | 0.176 | 0.000 | 0.167 | 0.833 | 0.833 |
| S1-57 | [337, 348] | 0.235 | 0.000 | 0.000 | 1.000 | 0.750 |
| S1-58 | [343, 354] | 0.118 | 0.000 | 0.083 | 0.917 | 0.667 |
| S1-59 | [349, 360] | 0.000 | 0.000 | 0.417 | 0.583 | 0.667 |
| *S1-60 | [355, 366] | 1.000 | 0.083 | 0.500 | 0.417 | 0.583 |
| S1-61 | [361, 372] | 0.000 | 0.333 | 0.167 | 0.500 | 0.583 |
| S1-62 | [367, 378] | 0.000 | 0.250 | 0.250 | 0.500 | 0.917 |
| S1-63 | [373, 384] | 0.353 | 0.000 | 0.333 | 0.667 | 0.833 |
| S1-64 | [379, 390] | 0.294 | 0.000 | 0.083 | 0.917 | 0.583 |
| *S1-65 | [385, 396] | 0.824 | 0.000 | 0.333 | 0.667 | 0.250 |
| S1-66 | [391, 402] | 0.412 | 0.000 | 0.833 | 0.167 | 0.000 |

| | | | | | | |
|---|---|---|---|---|---|---|
| S1-67 | [397, 408] | 0.471 | 0.000 | 0.500 | 0.500 | 0.306 |
| S1-68 | [403, 414] | 0.059 | 0.000 | 0.000 | 1.000 | 0.556 |
| S1-69 | [409, 420] | 0.294 | 0.000 | 0.000 | 1.000 | 0.583 |
| S1-70 | [415, 426] | 0.118 | 0.000 | 0.000 | 1.000 | 0.583 |
| S1-71 | [421, 432] | 0.000 | 0.000 | 0.167 | 0.833 | 0.583 |
| S1-72 | [427, 438] | 0.000 | 0.000 | 0.583 | 0.417 | 0.500 |
| S1-73 | [433, 444] | 0.059 | 0.000 | 0.417 | 0.583 | 0.500 |
| S1-74 | [439, 450] | 0.000 | 0.000 | 0.000 | 1.000 | 0.750 |
| S1-75 | [445, 456] | 0.000 | 0.000 | 0.333 | 0.667 | 0.750 |
| S1-76 | [451, 462] | 0.294 | 0.000 | 0.333 | 0.667 | 0.667 |
| S1-77 | [457, 468] | 0.176 | 0.000 | 0.000 | 1.000 | 0.778 |
| S1-78 | [463, 474] | 0.059 | 0.000 | 0.083 | 0.917 | 0.861 |
| S1-79 | [469, 480] | 0.059 | 0.000 | 0.083 | 0.917 | 0.917 |
| S1-80 | [475, 486] | 0.294 | 0.000 | 0.000 | 1.000 | 1.000 |
| S1-81 | [481, 492] | 0.059 | 0.000 | 0.167 | 0.833 | 0.917 |
| S1-82 | [487, 498] | 0.059 | 0.000 | 0.417 | 0.583 | 0.667 |
| S1-83 | [493, 504] | 0.059 | 0.167 | 0.250 | 0.583 | 0.750 |
| S1-84 | [499, 510] | 0.176 | 0.250 | 0.250 | 0.500 | 0.583 |
| S1-85 | [505, 516] | 0.471 | 0.083 | 0.667 | 0.250 | 0.167 |
| S1-86 | [511, 522] | 0.176 | 0.000 | 0.417 | 0.583 | 0.500 |
| S1-87 | [517, 528] | 0.176 | 0.000 | 0.167 | 0.833 | 0.583 |
| S1-88 | [523, 534] | 0.059 | 0.000 | 0.167 | 0.833 | 0.667 |
| S1-89 | [529, 540] | 0.118 | 0.000 | 0.083 | 0.917 | 0.833 |
| S1-90 | [535, 546] | 0.235 | 0.000 | 0.250 | 0.750 | 0.556 |
| *S1-91 | [541, 552] | 0.706 | 0.000 | 0.583 | 0.417 | 0.444 |
| S1-92 | [547, 558] | 0.235 | 0.000 | 0.583 | 0.417 | 0.722 |
| S1-93 | [553, 564] | 0.294 | 0.000 | 0.167 | 0.833 | 0.833 |
| *S1-94 | [559, 570] | 0.647 | 0.000 | 0.167 | 0.833 | 0.583 |
| S1-95 | [565, 576] | 0.059 | 0.000 | 0.333 | 0.667 | 0.500 |
| S1-96 | [571, 582] | 0.353 | 0.000 | 0.250 | 0.750 | 0.667 |
| *S1-97 | [577, 588] | 0.824 | 0.000 | 0.500 | 0.500 | 0.750 |
| S1-98 | [583, 594] | 0.118 | 0.000 | 0.417 | 0.583 | 0.417 |
| S1-99 | [589, 600] | 0.412 | 0.000 | 0.417 | 0.583 | 0.167 |
| S1-100 | [595, 606] | 0.353 | 0.000 | 0.417 | 0.583 | 0.500 |
| *S1-101 | [601, 612] | 1.000 | 0.000 | 0.167 | 0.833 | 0.583 |
| S1-102 | [607, 618] | 0.294 | 0.000 | 0.250 | 0.750 | 0.500 |
| S1-103 | [613, 624] | 0.176 | 0.333 | 0.083 | 0.583 | 0.500 |
| S1-104 | [619, 630] | 0.412 | 0.333 | 0.000 | 0.667 | 0.583 |
| *S1-105 | [625, 636] | 1.000 | 0.000 | 0.000 | 1.000 | 0.750 |
| *S1-106 | [631, 642] | 0.941 | 0.000 | 0.000 | 1.000 | 0.833 |
| S1-107 | [637, 648] | 0.000 | 0.000 | 0.333 | 0.667 | 0.833 |
| S1-108 | [643, 654] | 0.176 | 0.000 | 0.583 | 0.417 | 0.500 |
| S1-109 | [649, 660] | 0.176 | 0.000 | 0.333 | 0.667 | 0.667 |

| | | | | | | |
|---|---|---|---|---|---|---|
| S1-110 | [655, 666] | 0.294 | 0.000 | 0.250 | 0.750 | 0.917 |
| *S1-111 | [661, 672] | 0.882 | 0.000 | 0.417 | 0.583 | 0.500 |
| S1-112 | [667, 678] | 0.235 | 0.000 | 0.333 | 0.667 | 0.417 |
| S1-113 | [673, 684] | 0.471 | 0.000 | 0.083 | 0.917 | 0.833 |
| S1-114 | [679, 685] | 0.059 | 0.000 | 0.000 | 1.000 | 1.000 |
| S2-1 | [686, 697] | 0.000 | 0.000 | 0.333 | 0.667 | 0.500 |
| S2-2 | [692, 703] | 0.000 | 0.000 | 0.333 | 0.667 | 0.583 |
| S2-3 | [698, 709] | 0.059 | 0.000 | 0.000 | 1.000 | 1.000 |
| *S2-4 | [704, 715] | 0.706 | 0.000 | 0.417 | 0.583 | 0.667 |
| S2-5 | [710, 721] | 0.294 | 0.000 | 0.917 | 0.083 | 0.500 |
| S2-6 | [716, 727] | 0.000 | 0.000 | 1.000 | 0.000 | 0.750 |
| S2-7 | [722, 733] | 0.000 | 0.000 | 0.667 | 0.333 | 0.528 |
| S2-8 | [728, 739] | 0.412 | 0.167 | 0.417 | 0.417 | 0.278 |
| S2-9 | [734, 745] | 0.000 | 0.500 | 0.250 | 0.250 | 0.333 |
| S2-10 | [740, 751] | 0.059 | 0.667 | 0.000 | 0.333 | 0.583 |
| *S2-11 | [746, 757] | 1.000 | 0.333 | 0.000 | 0.667 | 0.750 |
| S2-12 | [752, 763] | 0.000 | 0.417 | 0.000 | 0.583 | 0.694 |
| S2-13 | [758, 769] | 0.000 | 0.917 | 0.000 | 0.083 | 0.778 |
| S2-14 | [764, 775] | 0.235 | 1.000 | 0.000 | 0.000 | 0.583 |
| *S2-15 | [770, 781] | 0.765 | 1.000 | 0.000 | 0.000 | 0.417 |
| S2-16 | [776, 787] | 0.235 | 0.583 | 0.000 | 0.417 | 0.583 |
| S2-17 | [782, 793] | 0.176 | 0.083 | 0.000 | 0.917 | 0.583 |
| S2-18 | [788, 799] | 0.294 | 0.000 | 0.083 | 0.917 | 0.667 |
| *S2-19 | [794, 805] | 0.706 | 0.000 | 0.167 | 0.833 | 0.583 |
| S2-20 | [800, 811] | 0.471 | 0.000 | 0.083 | 0.917 | 0.583 |
| S2-21 | [806, 817] | 0.471 | 0.083 | 0.000 | 0.917 | 0.833 |
| *S2-22 | [812, 823] | 0.824 | 0.583 | 0.000 | 0.417 | 0.583 |
| S2-23 | [818, 829] | 0.176 | 0.500 | 0.000 | 0.500 | 0.667 |
| S2-24 | [824, 835] | 0.000 | 0.333 | 0.000 | 0.667 | 0.806 |
| S2-25 | [830, 841] | 0.000 | 0.333 | 0.000 | 0.667 | 0.389 |
| S2-26 | [836, 847] | 0.235 | 0.000 | 0.000 | 1.000 | 0.583 |
| S2-27 | [842, 853] | 0.059 | 0.000 | 0.000 | 1.000 | 0.833 |
| S2-28 | [848, 859] | 0.176 | 0.000 | 0.167 | 0.833 | 0.528 |
| S2-29 | [854, 865] | 0.412 | 0.000 | 0.333 | 0.667 | 0.361 |
| S2-30 | [860, 871] | 0.235 | 0.417 | 0.167 | 0.417 | 0.250 |
| *S2-31 | [866, 877] | 0.588 | 0.917 | 0.000 | 0.083 | 0.167 |
| S2-32 | [872, 883] | 0.059 | 1.000 | 0.000 | 0.000 | 0.083 |
| S2-33 | [878, 889] | 0.059 | 0.500 | 0.000 | 0.500 | 0.222 |
| S2-34 | [884, 895] | 0.000 | 0.000 | 0.000 | 1.000 | 0.472 |
| S2-35 | [890, 901] | 0.000 | 0.333 | 0.000 | 0.667 | 0.333 |
| S2-36 | [896, 907] | 0.471 | 0.833 | 0.000 | 0.167 | 0.194 |
| S2-37 | [902, 913] | 0.000 | 0.750 | 0.000 | 0.250 | 0.444 |
| *S2-38 | [908, 919] | 0.529 | 0.667 | 0.000 | 0.333 | 0.500 |

| | | | | | | |
|---|---|---|---|---|---|---|
| S2-39 | [914, 925] | 0.059 | 0.917 | 0.000 | 0.083 | 0.500 |
| S2-40 | [920, 931] | 0.235 | 1.000 | 0.000 | 0.000 | 0.583 |
| S2-41 | [926, 937] | 0.118 | 1.000 | 0.000 | 0.000 | 0.667 |
| S2-42 | [932, 943] | 0.235 | 0.833 | 0.000 | 0.167 | 0.833 |
| S2-43 | [938, 949] | 0.000 | 0.833 | 0.000 | 0.167 | 0.722 |
| S2-44 | [944, 955] | 0.000 | 1.000 | 0.000 | 0.000 | 0.694 |
| *S2-45 | [950, 961] | 0.765 | 1.000 | 0.000 | 0.000 | 0.861 |
| S2-46 | [956, 967] | 0.353 | 0.750 | 0.000 | 0.250 | 0.639 |
| S2-47 | [962, 973] | 0.471 | 0.250 | 0.000 | 0.750 | 0.667 |
| S2-48 | [968, 979] | 0.353 | 0.250 | 0.000 | 0.750 | 0.917 |
| S2-49 | [974, 985] | 0.353 | 0.583 | 0.000 | 0.417 | 0.667 |
| S2-50 | [980, 991] | 0.176 | 0.833 | 0.000 | 0.167 | 0.667 |
| S2-51 | [986, 997] | 0.294 | 1.000 | 0.000 | 0.000 | 0.583 |
| S2-52 | [992, 1003] | 0.353 | 1.000 | 0.000 | 0.000 | 0.333 |
| S2-53 | [998, 1009] | 0.294 | 1.000 | 0.000 | 0.000 | 0.417 |
| S2-54 | [1004, 1015] | 0.353 | 1.000 | 0.000 | 0.000 | 0.500 |
| S2-55 | [1010, 1021] | 0.176 | 1.000 | 0.000 | 0.000 | 0.667 |
| *S2-56 | [1016, 1027] | 0.588 | 1.000 | 0.000 | 0.000 | 0.750 |
| S2-57 | [1022, 1033] | 0.000 | 1.000 | 0.000 | 0.000 | 0.333 |
| S2-58 | [1028, 1039] | 0.118 | 0.500 | 0.000 | 0.500 | 0.278 |
| S2-59 | [1034, 1045] | 0.000 | 0.000 | 0.000 | 1.000 | 0.583 |
| S2-60 | [1040, 1051] | 0.118 | 0.000 | 0.417 | 0.583 | 0.389 |
| S2-61 | [1046, 1057] | 0.000 | 0.000 | 0.833 | 0.167 | 0.083 |
| *S2-62 | [1052, 1063] | 0.765 | 0.000 | 0.833 | 0.167 | 0.000 |
| S2-63 | [1058, 1069] | 0.235 | 0.000 | 0.917 | 0.083 | 0.111 |
| S2-64 | [1064, 1075] | 0.118 | 0.000 | 1.000 | 0.000 | 0.528 |
| S2-65 | [1070, 1081] | 0.235 | 0.000 | 0.750 | 0.250 | 0.583 |
| S2-66 | [1076, 1087] | 0.176 | 0.000 | 0.583 | 0.417 | 0.417 |
| S2-67 | [1082, 1093] | 0.059 | 0.000 | 0.583 | 0.417 | 0.417 |
| S2-68 | [1088, 1099] | 0.176 | 0.000 | 0.583 | 0.417 | 0.417 |
| S2-69 | [1094, 1105] | 0.412 | 0.000 | 0.667 | 0.333 | 0.583 |
| S2-70 | [1100, 1111] | 0.235 | 0.000 | 0.333 | 0.667 | 0.667 |
| S2-71 | [1106, 1117] | 0.412 | 0.083 | 0.250 | 0.667 | 0.750 |
| S2-72 | [1112, 1123] | 0.176 | 0.250 | 0.583 | 0.167 | 0.750 |
| S2-73 | [1118, 1129] | 0.353 | 0.167 | 0.333 | 0.500 | 0.833 |
| S2-74 | [1124, 1135] | 0.000 | 0.000 | 0.083 | 0.917 | 1.000 |
| *S2-75 | [1130, 1141] | 1.000 | 0.000 | 0.167 | 0.833 | 1.000 |
| S2-76 | [1136, 1147] | 0.235 | 0.500 | 0.083 | 0.417 | 1.000 |
| S2-77 | [1142, 1153] | 0.118 | 1.000 | 0.000 | 0.000 | 0.750 |
| *S2-78 | [1148, 1159] | 1.000 | 0.750 | 0.000 | 0.250 | 0.583 |
| S2-79 | [1154, 1165] | 0.118 | 0.250 | 0.000 | 0.750 | 0.833 |
| S2-80 | [1160, 1171] | 0.059 | 0.000 | 0.000 | 1.000 | 1.000 |
| S2-81 | [1166, 1177] | 0.353 | 0.000 | 0.000 | 1.000 | 1.000 |

| | | | | | | |
|---|---|---|---|---|---|---|
| S2-82 | [1172, 1183] | 0.176 | 0.417 | 0.000 | 0.583 | 1.000 |
| S2-83 | [1178, 1189] | 0.412 | 0.917 | 0.000 | 0.083 | 1.000 |
| *S2-84 | [1184, 1195] | 0.588 | 1.000 | 0.000 | 0.000 | 0.917 |
| S2-85 | [1190, 1201] | 0.000 | 0.500 | 0.000 | 0.500 | 0.917 |
| *S2-86 | [1196, 1207] | 0.706 | 0.000 | 0.000 | 1.000 | 1.000 |
| *S2-87 | [1202, 1213] | 0.647 | 0.000 | 0.000 | 1.000 | 0 |
| *S2-88 | [1208, 1219] | 0.588 | 0.500 | 0.000 | 0.500 | 0 |
| S2-89 | [1214, 1225] | 0.000 | 1.000 | 0.000 | 0.000 | 0 |
| S2-90 | [1220, 1231] | 0.000 | 1.000 | 0.000 | 0.000 | 0 |
| S2-91 | [1226, 1237] | 0.000 | 0.583 | 0.000 | 0.417 | 0 |
| S2-92 | [1232, 1243] | 0.000 | 0.083 | 0.000 | 0.917 | 0 |
| S2-93 | [1238, 1249] | 0.000 | 0.000 | 0.000 | 1.000 | 0.833 |
| S2-94 | [1244, 1255] | 0.000 | 0.000 | 0.000 | 1.000 | 0.833 |
| S2-95 | [1250, 1261] | 0.118 | 0.000 | 0.000 | 1.000 | 0.917 |
| S2-96 | [1256, 1267] | 0.412 | 0.000 | 0.000 | 1.000 | 1.000 |
| S2-97 | [1262, 1273] | 0.412 | 0.000 | 0.000 | 1.000 | 1.000 |

**Supplementary Table 4** Data for the M50 S peptides covered the mutated amino acid residues of VOCs.

| **Domain** | **S Peptides** | **Mutation** | **VOCs lineage** |
|---|---|---|---|
| NTD | S1-3(aa:13-24) | L18F | P.1(Gamma) |
| NTD | S1-3(aa:13-24) | T19R | B.1.617.2(Delta) |
| NTD | S1-3(aa:13-24) | T20N | P.1(Gamma) |
| NTD | S1-27(aa:157-168) | R158G | B.1.617.2(Delta) |
| NTD | S1-31(aa:181-192) | R190S | P.1(Gamma) |
| NTD | S1-35(aa:205-216) | D215G | B.1.351(Beta) |
| RBD | S1-69(aa:409-420) | K417N | B.1.351(Beta); P.1(Gamma) |
| CTD1 | S1-94(aa:559-570) | A570D | B.1.1.7(Alpha); B.1.1.529(Omicron) |
| CTD2 | S1-113(aa:673-684) | N679K | B.1.1.529(Omicron) |
| CTD2 | S1-113(aa:673-684) | P681H | B.1.1.7(Alpha) |
| CTD2 | S1-113(aa:673-684) | P681R | B.1.617.2(Delta) |
| S2C1 | S2-19(aa:794-805) | D796Y | B.1.1.529(Omicron) |
| HR1 | S2-45(aa:950-961) | D950N | B.1.617.2(Delta) |
| HR1 | S2-45(aa:950-961) | Q954H | B.1.1.529(Omicron) |
| HR1 | S2-47(aa:962, 973) | N969K | B.1.1.529(Omicron) |
| CH | S2-56(aa:1016-1027) | T1027I | P.1(Gamma) |
| CT | S2-88(aa:1208-1219) | W1214G | B.1.1.7(Alpha) |
| NTD | S1-27(aa:157-168) | F157del | B.1.617.2(Delta) |
